# Bornyl Derivatives of *p*-(Benzyloxy)Phenylpropionic Acid: In Vivo Evaluation of Antidiabetic Activity

**DOI:** 10.3390/ph13110404

**Published:** 2020-11-19

**Authors:** Sergey Kuranov, Olga Luzina, Mikhail Khvostov, Dmitriy Baev, Darya Kuznetsova, Nataliya Zhukova, Pavel Vassiliev, Andrey Kochetkov, Tatyana Tolstikova, Nariman Salakhutdinov

**Affiliations:** 1 N. N. Vorozhtsov Institute of Organic Chemistry, Siberian Branch of Russian Academy of Sciences, 630090 Novosibirsk, Russia; s.o.kuranov@chemomsu.ru (S.K.); baev@nioch.nsc.ru (D.B.); dasha05-01-96@mail.ru (D.K.); gna2004@ngs.ru (N.Z.); tolstiktg@nioch.nsc.ru (T.T.); anvar@nioch.nsc.ru (N.S.); 2 Reasearch Center of Innovative Medicines, Laboratory for Information Technology in Pharmacology and Computer Modeling of Drugs, Volgograd State Medical University, Ministry of Health of Russian Federation, 400131 Volgograd, Russia; pvassiliev@mail.ru (P.V.); akocha@mail.ru (A.K.)

**Keywords:** type 2 diabetes mellitus, hypoglycemic activity, natural product, bornyl derivative, in silico prediction, hypoglycemic multitarget activity, glucose tolerance

## Abstract

A series of bornyl derivatives of *p*-(benzyloxy)phenylpropionic acid were prepared, and their hypoglycemic activities were examined by an oral glucose tolerance test in mice. The results of this test revealed two compounds, **1** and **3**, that can reduce the blood level of glucose similarly to reference compound vildagliptin. Both compounds were tested in an experiment on mice with metabolic disorders: the C57BL/6^Ay^ strain. Along with hypoglycemic properties, the two compounds showed different abilities to correct lipid metabolism disorders. In silico prediction revealed that the studied substances are most likely bifunctional multitarget hypoglycemic compounds whose mechanism of action is based on a pronounced reduction in insulin resistance and a strong incretin-mimetic effect. The difference in the size of effects of these compounds on biochemical parameters of blood in the experiment on C57BL/6^Ay^ mice was in good agreement with the computational prediction of the priority ranking of biological targets for these compounds. These results indicate that bornyl derivatives of *p*-(benzyloxy)phenylpropionic acid have a good potential as new agents for diabetes mellitus treatment due to their hypoglycemic and lipid-normalizing properties.

## 1. Introduction

Currently, global prevalence of diabetes mellitus among adults is 8.5%, and this figure is growing [1]. Diabetes mellitus is classified into type 1 diabetes mellitus and type 2 diabetes mellitus (T2DM), with the proportion of T2DM cases being almost 95%.

T2DM is a multisystem disease characterized by a disorder of many metabolic pathways, primarily carbohydrate metabolism. This disorder is caused by the body’s inability to use insulin to process glucose (i.e., insulin resistance). In the early stages of the disease, hyperglycemia contributes to the development of severe disturbances in insulin secretion. Uncompensated metabolism in T2DM leads to dyslipidemia with excessive concentrations of lipoproteins and lipids in the blood. Such metabolic disorders cause rapid progression of atherosclerosis and damage to the nervous system [2].

At T2DM diagnosis (usually accidental), patients already have complications of the disease in the form of visual impairment (retinopathy), kidney problems (micro- or macroproteinuria), and vascular lesions in the heart, brain, and lower extremities. The above complications are the main cause of death and high disability of T2DM patients. In addition, due to its chronicity, T2DM has become a costly disease for patients and their families as well as for the healthcare system [3,4]. Weight loss and lifestyle changes can partially suppress hyperglycemia [5], but antidiabetic drugs are needed to control glycemia and lipid levels [6]. The main adverse effects of modern oral antidiabetic agents include hypoglycemia, fluid retention, osteoporosis, and heart failure, which limit their clinical use. Therefore, the development of new antidiabetic candidate compounds for the control of various parameters of metabolic disturbances (hyperglycemia, hyperinsulinemia, and hypertriglyceridemia) with minimization of adverse effects remains the primary and most important task in the treatment of T2DM [7].

Medicinal preparations based on natural compounds are widespread in the pharmaceutical market [8] and often have a good safety profile and a milder (often prolonged) action and usually do not cause severe adverse effects and drug tolerance. Natural compounds are considered by researchers as potential therapeutic agents for diabetes mellitus [7]; however, only a small proportion of published research in the field of hypoglycemic agents is related to the targeted synthetic modification of natural metabolites.

In recent years, monoterpenoids have been reported to have antidiabetic effects. Various in vitro and in vivo data now suggest that despite their structural simplicity, monoterpenes hold promise as antidiabetic lead compounds either on their own or as part of structural moieties in complex compounds [9]. In particular, research has revealed that borneol has a beneficial effect on hyperglycemia along with antioxidant and antihyperlipidemic actions against streptozotocin-induced diabetes in rats in a dose-dependent manner [10].

Phenylpropanoic acid is a convenient structure for the synthesis of many targeted medicinal agents with hypoglycemic action [11]. In particular, substituted phenylpropanoic acids are agonists of receptors FFA4 [12], peroxisome proliferator-activated receptors (PPARs) [13] and protein tyrosine phosphatase 1B (PTP1B) inhibitors [14].

**Figure 1:**
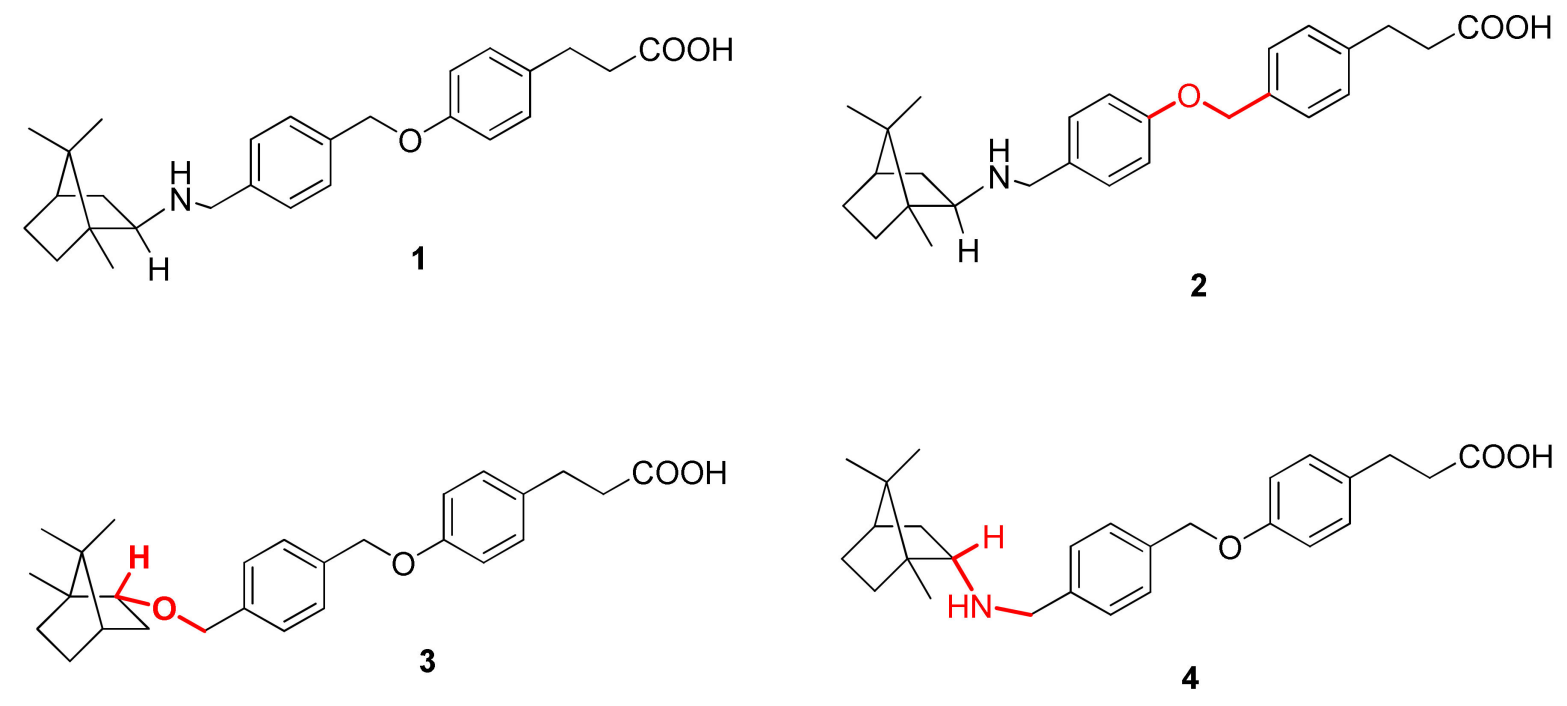
New bornyl derivatives.

Previously, we have synthesized bornyl derivative 1 (Figure 1) containing a pharmacophoric fragment of phenylpropanoic acid, for which a significant hypoglycemic effect—noninferior to that of reference compound vildagliptin (VIL)—was demonstrated in an oral glucose tolerance test (OGTT) performed on mice, and affinity for one of the targets of diabetes therapy (free fatty acid receptor 1) was confirmed [15]. 

The aim of the present study was to synthesize structural analogs of compound 1 and to test them in vivo to identify new compounds with hypoglycemic activity and to assess the structure–activity relationship. As in vivo models, we used two mouse strains: C57BL/6 for hypoglycemic-action screening by the OGTT and C57BL/6Ay for evaluation of selected compounds’ effects on animals with obesity and impaired glucose tolerance. In this case, the OGTT, blood biochemical assays, and liver histology examinations were performed. In silico prediction methods were used to explain differences in the action of structural analogs.

## 2. Results

### 2.1. Compound Selection

To determine the influence of structural features on biological activity, compound **1** analogs (**2**–**4**) with structural differences (Figure 1) were synthesized in this work. We synthesized derivative **2**, which features an unchangeable structure of a bornylamine substituent with an inverted structure of the oxymethylene linker. In a reaction with the natural compound borneol, derivative **3** was obtained in which a nitrogen atom was replaced by oxygen. As in compound **3,** the hydroxyl group of borneol is at the *endo*-position of the bornyl core in contrast to the *exo*-position of the amino group of bornylamine in compound **1**, we synthesized compound **4** (which shows the *endo*-configuration of the amino group) to reveal the impact of this center’s stereochemistry on biological activity. 

### 2.2. Synthesis

The synthesis of compound **1**, including the synthesis of key intermediate bromide **5**, has been described in our other study [15]. 

**Scheme 1:**
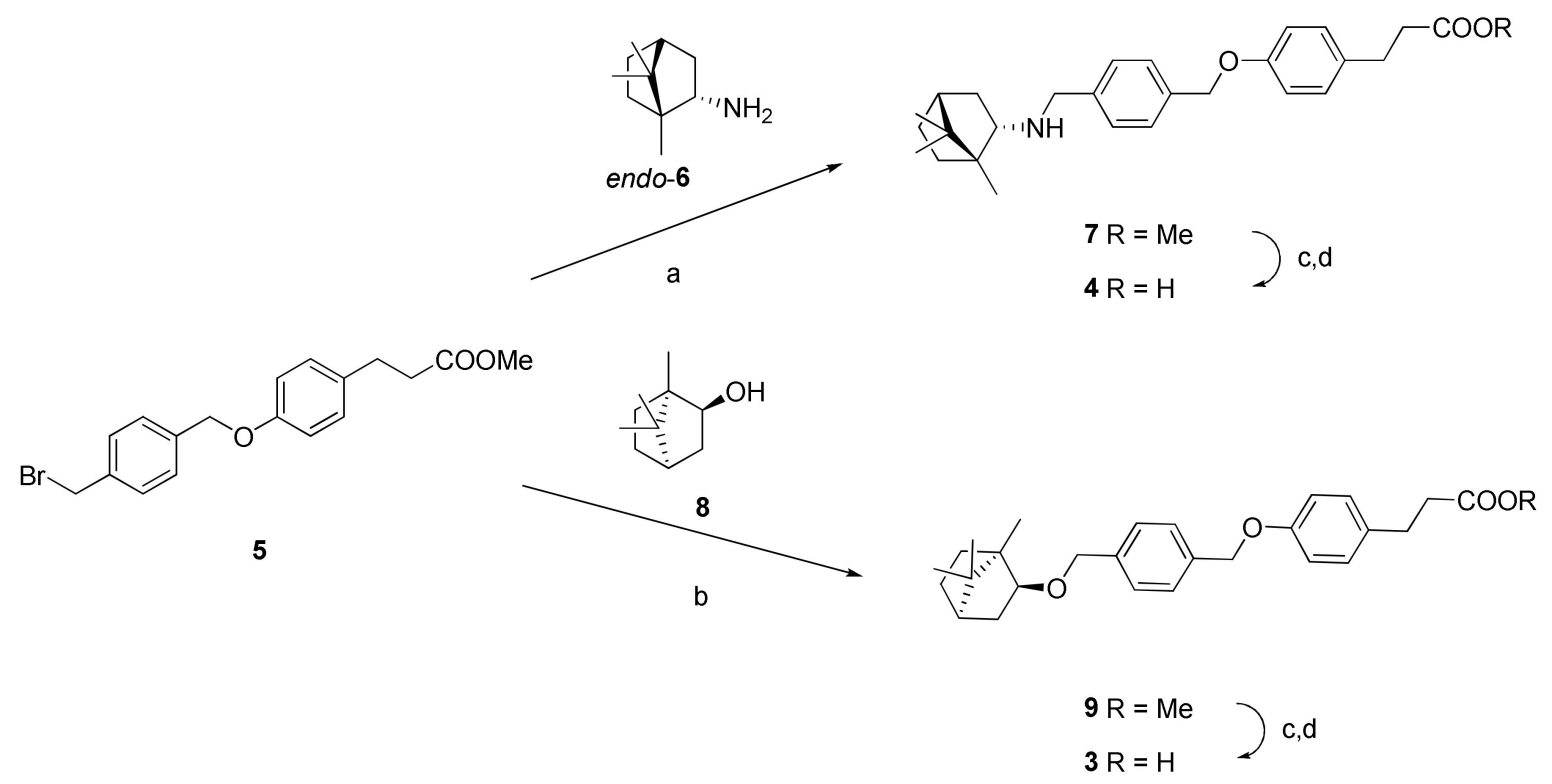
Reactions and conditions. **a**: *N,N*-diisopropylethylamine (DIPEA), CH_3_CN, reflux; **b**: DIPEA, potassium iodide, 150 °C; **c**: LiOH monohydrate, tetrahydrofuran (THF)/MeOH/H_2_O; **d**: HCl, H_2_O.

Bromide **5** was reacted with *endo*-bornylamine **6** when heated in acetonitrile in the presence of N,N-diisopropylethylamine (DIPEA; Scheme 1). Compound **7** (methyl ether) was isolated with a yield of 67% after chromatography. Sequential treatment of compound **7** with lithium hydroxide in a mixture of methanol–THF–water (4:2:3, where THF is tetrahydrofuran) and diluted hydrochloric acid led to the formation of compound **4** as hydrochloride with a yield of 52%.

To obtain compound **3**, bromide **5** was reacted with borneol **8** without a solvent in the presence of DIPEA and catalytic amounts of potassium iodide. Methyl ether **9** was isolated with a 41% yield after chromatography. Alkaline hydrolysis of methyl ether **9** was performed in the same way as described above; acid **3** was obtained in an 84% yield.

**Scheme 2:**
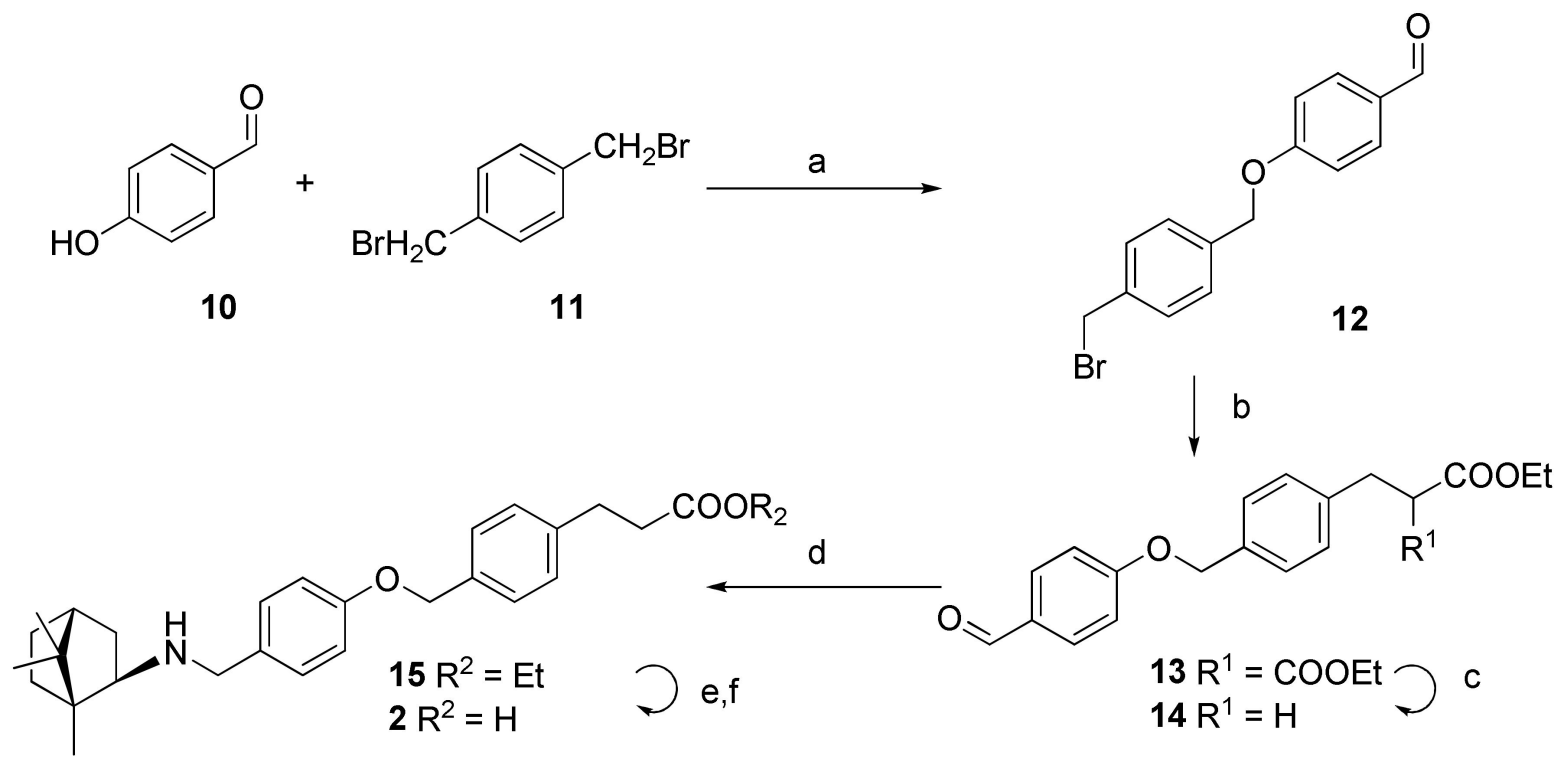
Reactions and conditions. a: K_2_CO_3_, acetone; b: CH_2_(COOEt)_2_, NaH, THF, 0 °C; c: NaCl, H_2_O, dimethyl sulfoxide (DMSO); d: *exo*-(1*R*)-**6**, NaBH(OAc)_3_, AcOH, CH_2_Cl_2_; e: LiOH monohydrate, THF/MeOH/H_2_O; f: HCl, H_2_O.

For the synthesis of compound **2** (it contains an inverse oxymethylene linker), we synthesized a new scaffold, **14** (Scheme 2). For this purpose, 4-hydroxybenzaldehyde **10** was reacted with 1,4-bis(bromomethyl)benzene **11** in acetone in the presence of potassium carbonate when heated, while bromaldehyde **12** formed with a yield of 62%. Resulting bromaldehyde **12** was reacted with diethylmalonate in THF in the presence of sodium hydride, and thus resulting in compound **13** with a yield of 67%. Krapcho decarboxylation of compound **13** with sodium chloride in aqueous dimethyl sulfoxide (DMSO) led to the formation of aldehyde **14** in a 78% yield. Reductive amination of aldehyde **14** with *exo*-bornylamine **6** led to amine **15** formation, hydrolysis of which by means of lithium hydroxide led to acid **2** in the form of hydrochloride with a yield of 44%. 

### 2.3. In Silico Analysis

For the synthesized compounds, we made a computer prediction of a profile of targeted hypoglycemic activities towards relevant biotargets. 

For comprehensive in silico evaluation of the compounds’ mechanism of hypoglycemic action, 24 key biotargets (Table 1) were chosen that had been verified in molecular pharmacology as important factors of diabetes pathogenesis [16,17,18].

Table 2 presents the indices of significance of the predicted biotargets, as calculated by a Microcosm BioS system [20,21] and reflecting the magnitude of the impact of the studied bornyl derivatives on these biotargets as well as structural similarity of these compounds to the nearest analogues from the quantitative structure–activity relationship (QSAR) base of standards.

To clarify the targeted nature of the antidiabetic activity of the studied compounds, they were docked into the binding sites of the three predicted most significant targets PPARD, FFAR1, and DPP4 (see Table 3).

Optimized 3D (three-dimensional) models of compounds were constructed by the methods of molecular mechanics and quantum chemistry sequentially in software MarvinSketch 17.1.23 [23] and MOPAC2016 [24].

Through a previously developed technique [25], three most valid experimental 3D models of the indicated *Homo sapiens* proteins were found: 3OZ0 for PPARD, 4PHU for FFAR1, and 3G0B for DPP4 [26].

Ensemble docking into specific sites of three valid models was performed using the AutoDock Vina 1.1.2 software [27] (10 conformers for each compound in 10 conformers five times in each site of each model, with the calculation of 50 obtained values of minimum docking energies ΔE).

A comparison of the calculated parameters of the level of targeted activity, obtained by the method of similarity to standards (Ind) and by the method of docking (ΔE), was performed by the entanglement method. This method, in contrast to correlation analysis (which reveals only linear continuous dependencies), allows the identification of any kind of relationship between two variables: linear or nonlinear, continuous or discrete.

In the present study, for each target k for each compound i, the value of each calculated indicator Ind_ki_ or ΔE_ki_ was converted into binary estimates, in accordance with their position relative to the mean value, according to the following rules:

1 if Ind_ki_ > mean (Ind_ki_), 0 otherwise;
1 if ΔE_ki_ < mean (ΔE_ki_), 0 otherwise.

A comparison of the two obtained series of binary estimates was performed via Spearman’s nonparametric correlation test.

The results of the analysis are shown in Table 4.

### 2.4. The OGTT in C57BL/6 Mice

**Figure 2:**
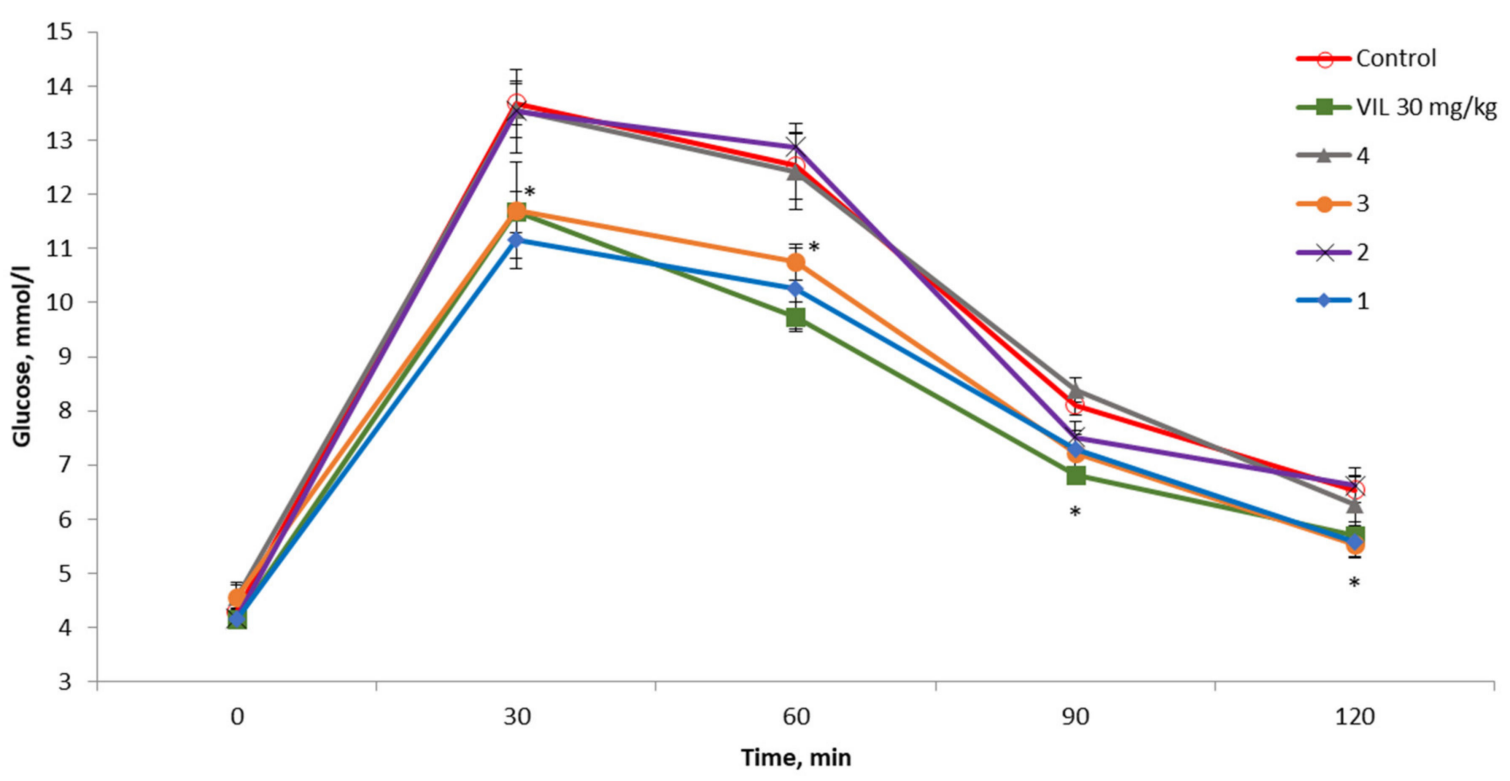
Oral glucose tolerance test (OGTT) results in C57BL/6 mice. All tested compounds (**1**–**4**) were administered at a dose of 30 mg/kg. * *p* < 0.05 as compared with untreated mice.

Compounds **1**–**4** were evaluated by the OGTT in a dose of 30 mg/kg. It was found that compounds **1** and **3** have hypoglycemic activity that is noninferior to the comparison compound VIL (Figure 2). These substances were selected for further evaluation in C57BL/6^Ay^ (hereafter: AY) mice.

### 2.5. The OGTT in AY Mice 

**Figure 3:**
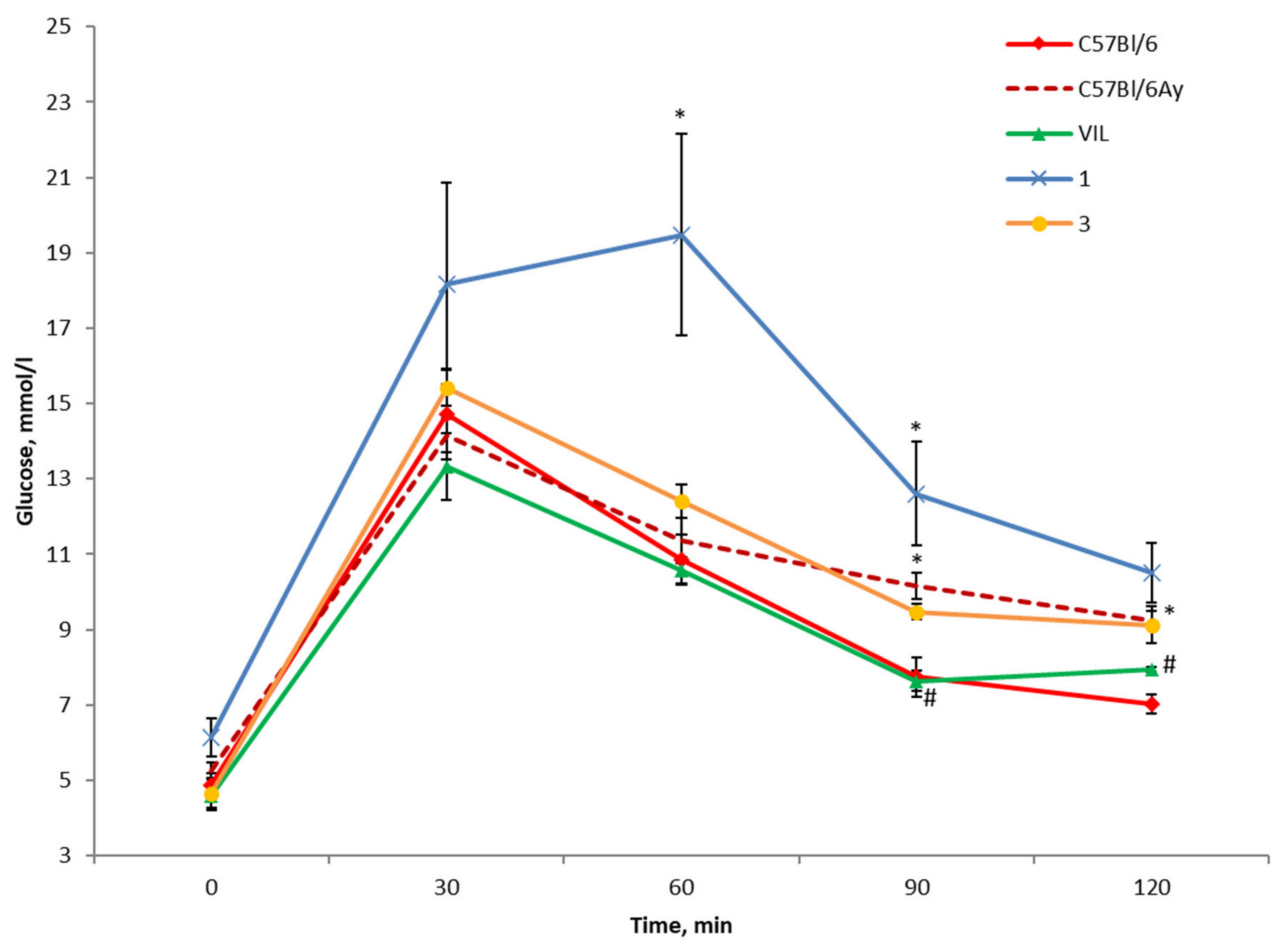
OGTT results in C57BL/6^Ay^ (AY) mice after the first administration of compound **1** or **3**. * *p* < 0.05 as compared with untreated C57BL/6 mice, ^#^ *p* < 0.05 as compared with untreated AY mice.

Compound **1** or **3** or a preparation of reference compound VIL was given intragastrically to male AY mice for 2 weeks. The mice were kept on a high-calorie diet for 2 months before the drug administration to accelerate obesity and to disturb glucose tolerance. After the first administration of a tested substance, the OGTT was immediately performed, and glucose tolerance was found to be impaired in AY mice (Figure 3), as evidenced by an increase in blood glucose levels at 90 and 120 min after the glucose administration as compared with untreated C57BL/6 mice.

The studied substances did not show a hypoglycemic effect. The glycemic profile after the administration of compound **3** virtually matched that of the untreated AY mice, whereas administration of compound **1** exerted a hyperglycemic effect by significantly increasing blood glucose concentration in these animals (Figure 3). In this experiment, a statistically significant decrease in blood glucose concentration was noted only in the positive control group (VIL; Figure 3). 

**Figure 4:**
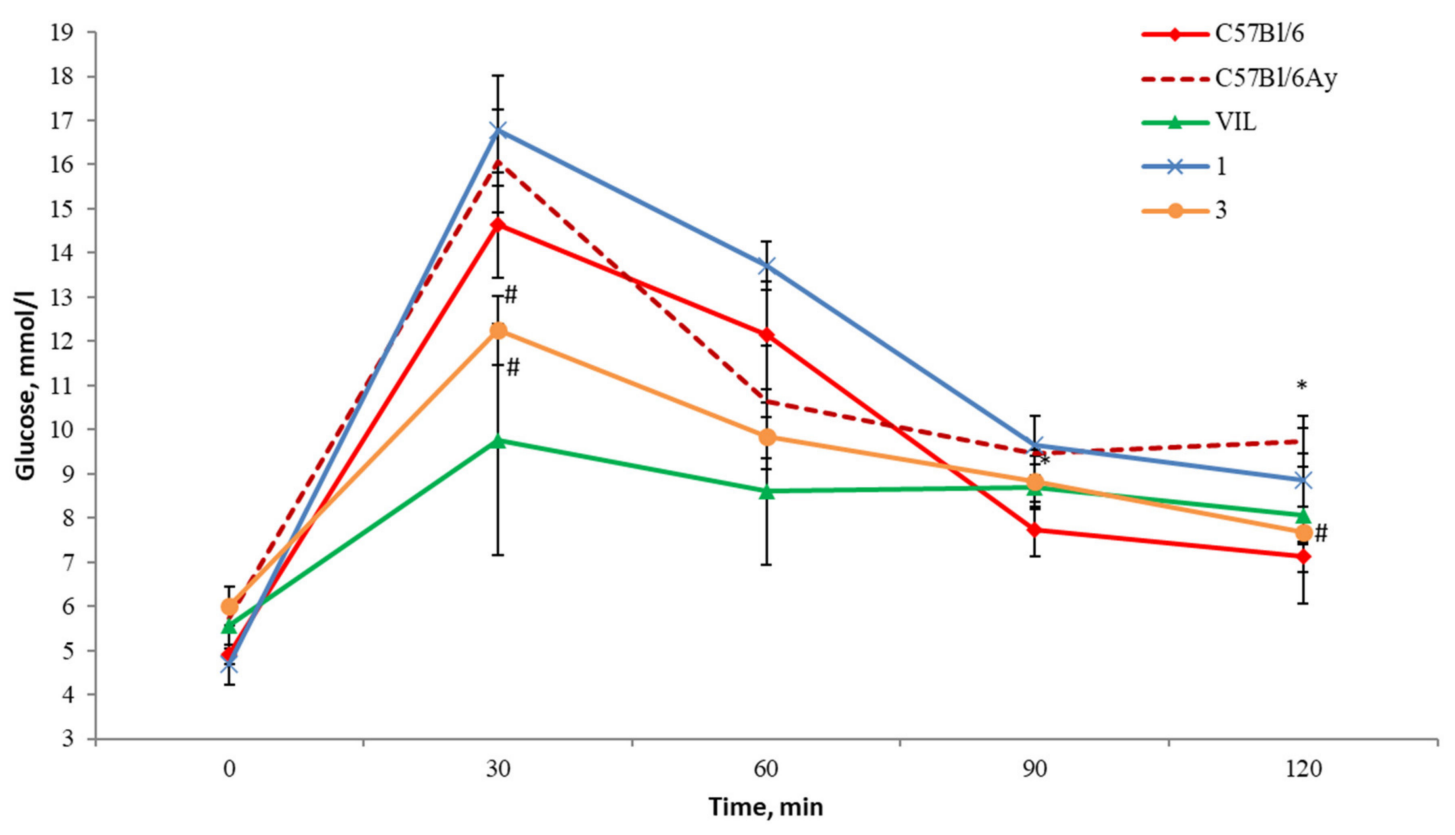
OGTT results in AY mice after 14 days of drug administration at a dose of 30 mg/kg. * *p* < 0.05 as compared with untreated C57BL/6 mice, ^#^ *p* < 0.05 as compared with untreated AY mice.

After 14 days of administration, another OGTT was carried out, where it was found that glucose tolerance in untreated AY mice (Figure 4) remained abnormal.

**Figure 5:**
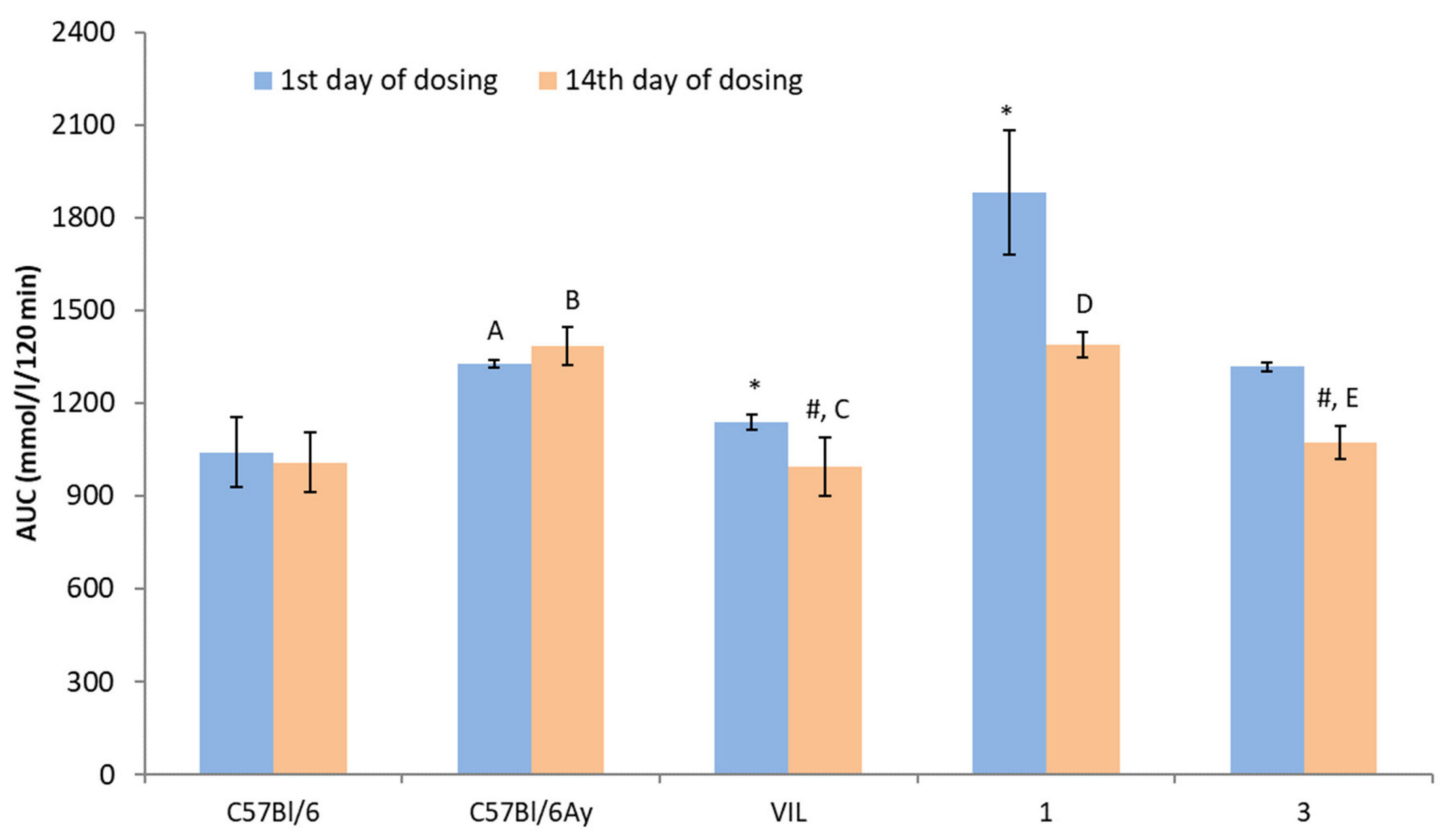
OGTT results in AY mice. AUC: area under the glycemic curve; * *p* < 0.05 vs. untreated AY mice on the first day of dosing; ^#^ *p* < 0.05 vs. untreated AY mice on the 14th day of dosing; ^A^ *p* < 0.05 vs. untreated C57BL/6 mice on the first day of dosing; ^B^ *p* < 0.05 vs. untreated C57BL/6 mice on the 14th day of dosing; ^C^ *p* < 0.05 vs. vildagliptin (VIL) on the first day of dosing; ^D^ *p* < 0.05 vs. compound **1** on the first day of dosing; ^E^ *p* < 0.05 vs. **3** on the first day of dosing.

At the same time, a significant hypoglycemic effect was detected in group three (treatment with compound **3**), judging by a lower glycemic curve (Figure 4) and area under the glycemic curve (AUC) (Figure 5). The hyperglycemic effect of compound **1** decreased after the 2 week administration, but the total glucose level in these animals did not differ from that in the untreated AY group (Figure 5). The administration of VIL for 2 weeks also improved the results of the OGTT; there was a significant decrease in the blood glucose level in this group.

### 2.6. A Biochemical Blood Assay in Treated AY Mice

Two days after the last OGTT, the animals were decapitated, and blood was collected for biochemical testing.

Table 5 shows that compound **1** decreased cholesterol concentration in the blood of AY mice, according to a reduction in total cholesterol and high-density lipoprotein cholesterol; in addition, there was a significant decrease in blood glucose concentration. Compound **3** did not show such an effect. The biochemical profile of AY mice in the VIL group was generally similar to that in the untreated AY group.

### 2.7. Histological Examination 

**Figure 6:**
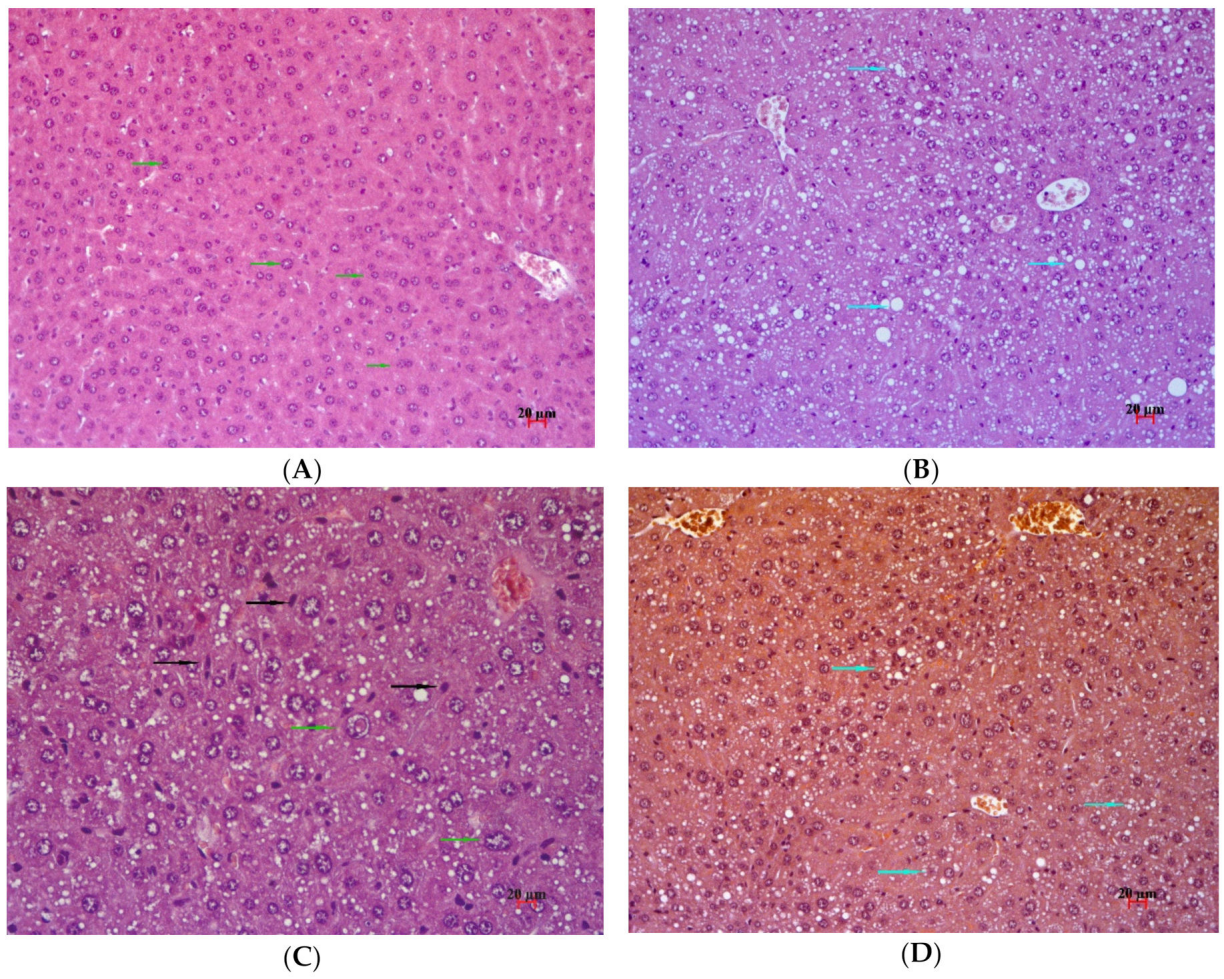
Histological examination of the liver of аn untreated C57BL/6 mouse (**A**) and an untreated AY mouse (**B**–**D**) at the end of the experiment. A. Nuclear polymorphism of hepatocytes. Staining with hematoxylin and eosin, magnification ×200. (**B**) Polymorphic lipid infiltration of hepatocytes. Staining with hematoxylin and eosin; magnification ×200. (**C**). Polymorphic lipid infiltration, nuclear polymorphism of hepatocytes. Staining with hematoxylin and eosin; magnification ×400. (**D**) Polymorphic lipid infiltration, no glycogen. Staining: periodic acid–Schiff, hematoxylin, and orange G; magnification ×200. Light blue arrow: lipid infiltration, black arrow: Kupffer cell, green arrow: nuclear polymorphism.

At the end of the experiment, the liver was collected from the mice for histological examination. In untreated C57BL/6 mice, liver architectonics were found to be preserved; bile capillaries, veins, and arteries had typical structure, and there were no signs of pathological infiltration, necrosis, and fibrosis (Figure 6A). Dystrophic changes in the form of focal cytoplasm disruption were registered in hepatocytes of periportal zones, and nuclear polymorphism was often observed in hepatocytes. In sinusoids, Kupffer cells without signs of increased activity and stand-alone mononuclear leukocytes were detectable.

In the liver of AY mice in the untreated group, after 14 days, typical signs of damage were observed in the form of pronounced polymorphic lipid infiltration of hepatocytes (Figure 6B). All animals in this group showed signs of an increase in the regenerative activity of the liver, as evidenced by slight differences in nuclei and in hepatocyte size (different sizes of hepatocytes, nuclei, and intranuclear inclusions) and activated Kupffer cells (Figure 6C). Glycogen was not detectable in hepatocytes (Figure 6D).

**Figure 7:**
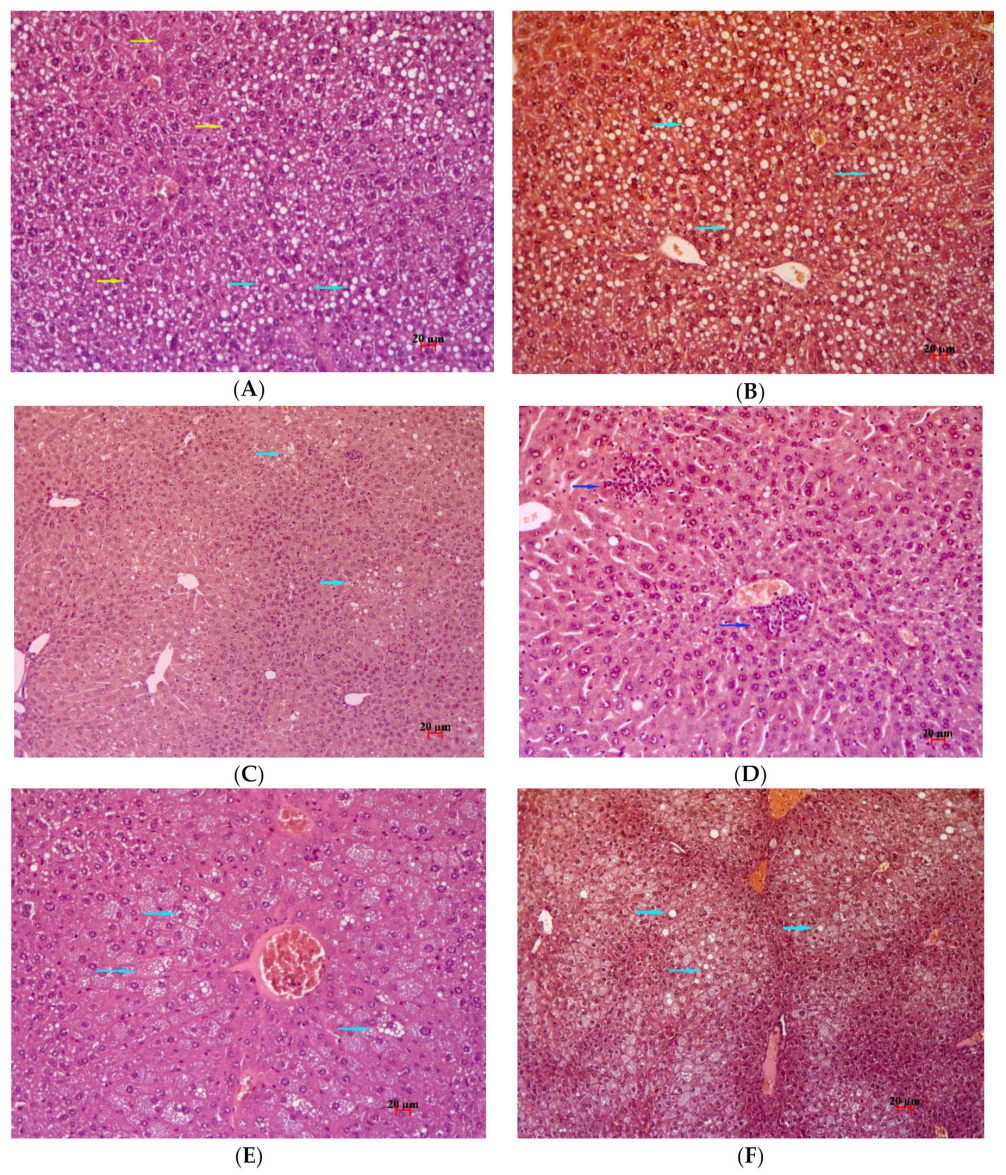
Histological examination of the liver of AY mice treated with VIL or compound **1** or **3**. (**A**) The liver of an AY mouse after 14 days of VIL administration. Polymorphic lipid infiltration and hepatocyte polymorphism. Staining: hematoxylin and eosin; magnification ×200. (**B**) The liver of an AY mouse after 14 days of administration of VIL. Polymorphic lipid infiltration, no glycogen. Staining: periodic acid–Schiff, hematoxylin, and orange G; magnification ×200. (**C**) The liver of an AY mouse that received 1 for 14 days. Polymorphic lipid infiltration, no glycogen. Staining: periodic acid–Schiff, hematoxylin, and orange G; magnification ×200. (**D**) The liver of an AY mouse that received **1** for 14 days. Fine necrosis of hepatocytes. Staining: hematoxylin and eosin; magnification ×200. (**E**) The liver of an AY mouse who received **3** for 14 days. Small-vesicle fat dystrophy, hepatocyte hypertrophy. Staining with hematoxylin and eosin; magnification ×200. (**F**) The liver of an AY mouse that received **3** for 14 days. Small-vesicle fat dystrophy, no glycogen. Staining: periodic acid–Schiff, hematoxylin, orange G; magnification ×200. Light blue arrow: lipid infiltration, yellow arrow: polymorphic hepatocytes, blue arrow: necrosis.

In AY mice treated with VIL, no obvious signs of normalization of metabolic disturbances were observed. The prime sign of liver damage was well-pronounced polymorphic infiltration of hepatocytes with cellular and nuclear polymorphism (Figure 7A). In contrast to the untreated group of AY mice, fatty degeneration was localized mainly to the central zones of lobules. Activated Kupffer cells were found in sinusoids. Glycogen was not detectable in hepatocytes (Figure 7B).

In AY mice that received **1** for 14 days, there was alleviation of metabolic disturbances. Only one animal in this group manifested small-vesicle fat dystrophy (in small foci), which was localized to the central sections of lobules, and necrosis of hepatocytes in small foci was observed (Figure 7C). In other animals of this group, there were no obvious signs of metabolic disturbances. Activated Kupffer cells were found in sinusoids. Glycogen in hepatocytes was not detectable (Figure 7D).

AY mice treated with **3** for 14 days showed partial correction of the metabolic abnormalities. Fatty degeneration in this group was more pronounced than in the animals that received **1** for 14 days (Figure 7E). For the most part, small-vesicle fatty degeneration was observed in these mice. It should be noted that in all animals of this group, there was an increase in regenerative activity in the form of hypertrophy of hepatocytes with nuclear polymorphism. In sinusoids, enlarged Kupffer cells were seen. Additionally, a narrowing of sinusoidal lumens was visible, which was associated with hypertrophy of hepatocytes (Figure 7F). No glycogen was detectable in hepatocytes. 

## 3. Discussion

In this study, we synthesized new bornyl derivatives of *p*-(benzyloxy)phenylpropionic acid. The new compounds have structural features that distinguish them from previously obtained compound **1**, which manifested a hypoglycemic effect that is noninferior to the reference drug VIL in experiments on animal models [15]. Compound **2** has an inverted oxymethylene linker structure; in compound **3**, the terpenoid fragment is linked to the *p*-(benzyloxy)phenylpropionic acid moiety by an oxygen atom instead of nitrogen. In a stereoisomer of compound **1**, i.e., acid **4**, the amino group of the bornyl part is located at the *endo*-position, in contrast to the *exo*-arrangement of the amino group in compound **1**.

The evaluation of the synthesized compounds in the OGTT performed on C57BL/6 mice at a dose of 30 mg/kg showed that only compounds **1** and **3** have hypoglycemic activity. Both of them reduced blood glucose levels to ~15% at each time point after the glucose load. The size of the observed effect of these compounds was noninferior to that of the reference drug VIL. The obtained data indicate that both the change in the oxymethylene linker structure and the configuration of the amino group in the bornyl substituent have a significant influence on the hypoglycemic properties of these compounds.

Compounds **1** and **3** were selected for further characterization in an experiment on AY mice. These mice carry an autosomal spontaneous yellow mutation at the *agouti* locus (*A^y^* mice), which causes uncontrolled and ubiquitous expression of the *Agouti* gene. The gene product, the Agouti protein, is a natural antagonist of melanocortin receptors, i.e., the mutation reduces the activity of melanocortin receptors in all cells of the body, thereby causing an increase in appetite and a decrease in energy expenditure. With age, AY mice develop obesity, T2DM, hyperglycemia, and hyperinsulinemia [28].

Our experiments on AY mice uncovered obvious differences in effects between compounds **1** and **3**. The first administration of **3** caused no effect on the glycemic curve in the OGTT test. The 14 day administration improved glucose uptake, most likely due to a decrease in insulin resistance because the effect materialized only after 2 weeks of administration. Liver histological examination revealed alleviation (by treatment with **3**) of the fatty degeneration but it was less pronounced than that of compound **1.** By contrast, **1** after the first administration to AY mice caused pronounced elevation of the blood glucose level in the OGTT; this hyperglycemic effect significantly diminished after 14 days of treatment with **1** along with prominent liver function recovery. 

To clarify the difference in the biological action between the studied compounds, the profiles of effects of the new bornyl derivatives on biotargets relevant to hypoglycemic properties were analyzed by in silico methods.

According to the in silico data (Table 2), the studied compounds have a significant effect on 12 out of the 24 analyzed biotargets. The average index of the activity level, Ind_Mean_, was ≥ 0 (not lower than the average), which indicates a pronounced multitargeting effect of these compounds. Eight of these 12 significant targets can be regarded as highly significant because Ind_Mean_ was ≥1 (activity above average). Among these, one biotarget, PPARδ, is very highly significant, i.e., the bornyl derivatives have high activity towards this target.

It should be noted that high similarity coefficients, T_Mean_ ≥ 0.4 (highlighted in bold in Table 2), support the validity of the calculated indices of the activity level. In this context, the top six highly significant targets—PPARδ, FFAR1, DPP4, PYGL, PTPN1, and FFAR4—can be considered especially interesting.

A comparison of the data between Table 1 and Table 2 suggests that, in general, the studied bornyl derivatives exert a bifunctional multitarget effect with high significance; mainly, they decrease insulin resistance (five significant targets: PPARδ, FFAR1, PTPN1, FFAR4, and PPARα) and also have properties of incretin-mimetics (three significant targets: DPP4, GIPR, and GLP1R).

The sum of indices of the activity level for significant targets affecting insulin resistance and having the effect of incretin-mimetics, Σ Ind_Mean_, was 10.50, whereas the sum for 12 significant targets, Σ Ind_Mean_, was 14.77. This means that for the bornyl derivatives, this well-pronounced combined activity is the main multitarget mechanism behind their hypoglycemic effect.

It is also possible that the studied substances influence the processes of glycolysis and glycogen synthesis (two significant targets: PDK4 and PFKM), gluconeogenesis and glycogenolysis (one significant target: PYGL), and the degradation of poly- and oligosaccharides (one significant target: AMY2A), but these are minor multitarget hypoglycemic properties of the bornyl derivatives.

Compound **3** has 17 significant biotargets in the range of activity Ind_Mean_ ≥ 0, among which 12 are very significant, with Ind_Mean_ ≥ 1; the other three compounds have 12 and 8 such biotargets, respectively. Compound **3** is the only molecule for which there are two biotargets, PPARD and FFAR1, with Ind_Mean_ > 3 (very highly active).

The sum of indices of the activity level for all biotargets, Σ Ind_Mean_, for compound **3** is 16.21, whereas for the other three compounds, this value ranges from 0.12 to −1.40. At the same time, for compound **3**, the sum of indices of the activity level, Σ Ind_Mean_, for significant biotargets is 24.36, and for the other three structures, this index is in the range 14.21 to 15.36.

Among the 17 biotargets significant for compound **3**, six affect insulin resistance and four cause incretin-mimetic actions. Thus, compound **3** can be considered a bifunctional multitarget hypoglycemic agent whose mechanism of action can be based on a pronounced ability to reduce insulin resistance and a strong incretin-mimetic effect.

A nonparametric analysis of the correlation of the two predictive parameters obtained by the method of similarity to standards (Ind) and by the docking method (ΔE) showed that there is a statistically highly significant interdependence between them (*p* = 9.06 × 10^−3^).

Thus, by the docking method, the present authors show the validity of the estimates obtained by the method of similarity to standards. Vice versa, the validity of the calculated estimates obtained by the docking method was confirmed by the results of the prediction by the method of similarity to standards. It should be emphasized that the calculated estimates of the effect on three significant biotargets obtained by the two methods for active substance **3** correlate very well:

– For PPARD, the highest Ind value corresponds to the highest docking energy ΔE;
– For FFAR1 also, the highest value of the Ind estimate corresponds to the highest docking energy ΔE;
– For DPP4, the lowest value of the Ind estimate corresponds to the minimum docking energy ΔE.

Therefore, the results on the prediction of targeted activity indicate a high probability of interaction of compound **3** with a large number of biotargets responsible for a decrease in insulin resistance: peroxisome proliferator-activated receptor delta, free fatty acid receptor 1 (GPR40), peroxisome proliferator-activated receptor gamma, free fatty acid receptor 2 (GPR43), protein-tyrosine phosphatase 1B (PTP1B), and free fatty acid receptor 4 (GPR120). These calculation results are consistent with the less pronounced alleviation (compared with compound **1**) of fatty degeneration of the liver during the administration of **3.** The latter observation suggests that the liver condition improvement in these mice is due to the decrease in insulin resistance caused by **1** but is not a key factor leading to glucose metabolism improvement. The hypoglycemic effect seen in the OGTT during the selection of a lead compound in the experiment on C57BL/6 mice (without T2DM and without concomitant insulin resistance) is most likely due to the action of **3** on free fatty acid receptor 1 (GPR40), trace amine-associated receptor 1, and to a lesser extent dipeptidyl peptidase 4 [29,30]. The lack of effect after the first administration of **3** to AY mice is probably due to insufficient stimulation of the insulin release for overcoming insulin resistance in these mice [31]. According to the results of our biochemical assays, the fasting glucose level after 14 days of administration of **3** also tends to decrease, indicating the need for longer administration of this substance to obtain a reliable effect.

On the contrary, the predicted activity of compound **1** is more indicative of its incretin-mimetic effect because of the inhibition of DPP4 and activation of GPR40, while actions on the targets responsible for a decrease in insulin resistance are much less likely. These data can explain the effect of **1** in the OGTT performed on normal mice free of T2DM and of insulin resistance [15]. Its hyperglycemic action in the OGTT in AY mice may be mediated by the action of glucose-dependent insulinotropic polypeptide (GIP), which is a DPP4 substrate and its concentration increases when DPP4 is inhibited [30]. It is known that GIP not only stimulates the secretion of insulin but also promotes the release of glucagon, which induces an increase in glucose production in the liver. During DPP4 deficiency (inhibition or a knockout in mice), the sensitivity of pancreatic β-cells to this hormone may diminish, and as a result, insulin secretion may decrease, which, along with the action of glucagon and fatty liver disease, raises blood glucose levels [32]. In addition, it is known that the effect of VIL, a selective DPP4 inhibitor, can decrease with increasing doses; this phenomenon is also related to stronger inhibition of the enzyme not only in blood plasma but also, for example, in the wall of blood vessels of the pancreas [33]. The reason for this is that DPP4 that is dissolved in the blood may originate from endothelial or epithelial cells and from circulating leukocytes [32]. The observed decrease in this effect after 14 days of treatment with **1** is most likely associated with attenuated severity of fatty degeneration of the liver; this change can normalize liver function, restore insulin sensitivity, and normalize lipid metabolism. The mechanism underlying this effect may be partially due to PPAR-α activation as well as to actions on other targets that have not been evaluated in this work. In our experiment, VIL at a dose of 30 mg/kg inhibited DPP4 apparently only in blood plasma, and this phenomenon did not lead to DPP4 deficiency and the above-mentioned compensatory effect of GIP. The 14 day administration of VIL, in contrast to compounds **1** and **3**, had no impact on fatty degeneration of the liver in AY mice, and thus once again confirming the multitarget effect of **1** and **3**. In the future, it is necessary to conduct additional experiments for elucidating the mechanisms of action of **1** and **3** including measurement of insulin and levels of *in silico*-predicted target proteins.

## 4. Materials and Methods

### 4.1. Chemistry

^1^H and ^13^C NMR spectra were acquired on Bruker spectrometers AV-400 at 400.13 MHz (^1^H) and 100.61 MHz (^13^C), AV-600 at 600.30 MHz (^1^H) and 150.95 MHz (^13^C) in deuterated chloroform (CDCl_3_) or deuterated dimethyl sulfoxide (DMSO-*d_6)_*; chemical shifts δ in parts per million (ppm) were measured relative to residual CHCl_3_ [δ(CHCl_3_) 7.26, δ(CHCl_3_) 77.00 ppm] or DMSO-*d_6_* [δ(DMSO-*d_6_*) 2.50, δ(DMSO-*d_6_*) 39.51 ppm], and *J* was measured in Hertz. The attached proton test (APT) modification of the J-modulated spin-echo (JMOD) experiment was performed. The structure of the products was determined by means of ^1^H and ^13^C NMR spectra. Optical rotation was measured on a polar 3005 spectrometer (CHCl_3_ solution). High-resolution mass spectrometry (HRMS) was conducted using a DFS Thermo Scientific spectrometer in full scan mode (*m/z* 15–500, 70 eV electron impact ionization, and direct sample injection). Column chromatography was performed on silica gel (60–200 mesh, Macherey-Nagel). Infrared spectra (IR) were measured on a Vector 22 FTIR spectrometer in potassium bromide (KBr) pellets. All the compounds reported in this paper had purity of at least 95%. Spectral and analytical measurements were carried out at the Multi-Access Chemical Service Center of Siberian Branch of Russian Academy of Sciences (SB RAS). 

*Exo*-bornylamine (*exo*-**6**), bromide **5**, and compound **1** were synthesized according to the procedure described previously [9]. *Endo*-bornylamine (*endo***-6**) was isolated as a minor isomer during *exo*-bornylamine purification by column chromatography. The structure of the obtained compounds was established in an analysis of the NMR spectra in accordance with ref. [34].

NMR spectra for compounds **2-4** and **12-15** are presented in the Supplementary (Figure S1–S13).

All reagents were analytically pure and were used as received without purification. In addition, 1,4-Bis(bromomethyl)benzene **11** (97%) and sodium hydride (60% dispersion in mineral oil) were bought from Acros organics. DIPEA and 4-hydroxybenzaldehyde were acquired from Carl Roth^®®^. Borneol was purchased from the Pilot Plant Department of the Novosibirsk Institute of Organic Chemistry SB RAS. Solvents were distilled prior to use: THF by distillation from sodium/benzophenone under nitrogen, whereas dichloromethane and acetonitrile were distilled from phosphorus pentoxide.

#### 4.1.1. General Procedure for Hydrolysis 

To a stirred mixture of ester (1.0 mmol) in 0.5 mL of methanol, 1.0 mL of THF, and 1.0 mL of water, we added solid LiOH monohydrate (2.2 mmol) at 0 °С. The reaction mixture was stirred at room temperature for 4 h. Methanol and THF were distilled off in vacuum, 5 mL of water was added to the residue, and 2 M hydrochloric acid was added with cooling to adjust pH to 2–3. The precipitate was filtered off and washed with distilled water.

#### 4.1.2. 3-(4-((4-(((1*R*,2*R*,4*R*)-1,7,7-Trimethylbicyclo[2.2.1]heptan-2-ylamino)methyl)- phenoxy)methyl)phenyl)propanoic acid hydrochloride **2**

Acid **2** was synthesized from ester **15**. White solid, yield 44%. Melting point (m.p.) (decomposition) 154.2 °C. [α]_D_^27^ −41 (c 0.147, EtOH). HRMS for C_27_H_35_O_3_N^+^ *m/z* calcd. [M]^+^ 421.2612, found 421.2617. IR (KBr, cm^−1^): ν 833, 1012, 1250, 1383, 1419, 1516, 1612, 1707. ^1^H NMR (300 MHz, DMSO-d_6_): δ 0.77 (3Н, s), 0.85–1.07 (8H, m), 1.39–1.76 (4H, m), 2.04 (2H, d, *J =* 9.5), 2.50–2.60 (2Н, t, *J =* 7.5), 2.77–2.91 (3Н, m), 3.90–4.09 (2Н, d), 5.02–5.12 (2Н, s), 7.02 (2Н, d, *J =* 8.3), 7.19–7.28 (2Н, d), 7.34 (2H, d, *J =* 7.9), 7.55 (2H, d, *J =* 7.8), 8.35–9.98 (2Н, br.s, NH_2_^+^). ^13^C NMR (101 MHz, DMSO-d_6_): δ 11.9, 20.1, 20.4, 26.5, 30.1, 33.7, 35.2, 36.3, 44.4, 46.7, 48.4, 50.3, 64.2, 69.0, 114.7 (2C), 127.9 (2C), 128.3 (2C), 131.5 (2C), 134.6, 140.6, 158.1, 173.8. Found, %: Cl 7.82. C_27_H_36_O_3_NCl. Calcd, %: Cl 7.74.

#### 4.1.3. 3-(4-(4-(((1*S*,2*R*,4*R*)-1,7,7-Trimethylbicyclo[2.2.1]heptan-2-yloxy)methyl)- benzyloxy)phenyl)propanoic acid **3**

Acid **3** was synthesized from ester **9**. White solid, yield 65%. M.p. (decomposition) 136.7 °C. [α]_D_^25^ −25 (c 0.130, CHCl_3_). HRMS for C_27_H_34_O_4_^+^ *m/z* calcd. [M]^+^ 422.2452, found 422.2456. IR (KBr, cm^−1^): ν 810, 827, 1124, 1248, 1514, 1707. ^1^H NMR (400 MHz, CDCl_3_): δ 0.81–0.94 (9Н, m), 1.10 (1H, d.d, *J =* 13.0, 3.2), 1.19–1.31 (2Н, m), 1.62–1.77 (2Н, m), 2.04–2.19 (2Н, m), 2.61–2.69 (2Н, t, *J*= 7.6), 2.90 (2H, t, *J =* 7.7), 3.66–3.74 (1Н, m), 4.42–4.49 (1Н, m), 4.55–4.62 (1Н, m), 5.03 (2Н, s), 6.91 (2H, d, *J =* 8.5), 7.13 (2H, d, *J =* 8.5), 7.33–7.43 (4Н, m). ^13^C NMR (75 MHz, CDCl_3_): δ 14.0, 18.9, 19.8 (2C), 26.8, 28.3, 29.7, 35.7, 36.1, 45.0, 47.9, 49.3, 69.9, 71.2, 84.4, 114.9 (2C), 127.4 (2C), 127.4 (2C), 129.2 (2C), 132.5, 135.9, 139.3, 157.4, 178.4.

#### 4.1.4. 3-(4-(4-(((1*R*,2*S*,4*R*)-1,7,7-Trimethylbicyclo[2.2.1]heptan-2-ylamino)methyl)- benzyloxy)phenyl)propanoic acid hydrochloride **4**

Acid **4** was synthesized from ester **7**. White solid, yield 55%. [α]_D_^25^ +19 (c 0.308, CHCl_3_). HRMS for C_27_H_34_O_4_^+^ *m/z* calcd. [M]^+^ 421.2612, found 421.2610. ^1^H NMR (400 MHz, DMSO-*d_6_*): δ 0.70 (3H, s), 0.80 (3H, s), 0.88 (3H, s), 1.21 (1H, m), 1.33–1.49 (2H, m), 1.60–1.76 (3H, m), 1.87–1.99 (1H, m), 2.44–2.54 (2H, m), 2.73 (2H, t, *J =* 7.1), 3.08 (1H, br. s.), 4.04–4.25 (2H, m), 5.10 (2H, br. s.), 6.90 (2H, d, *J =* 7.9), 7.12 (2H, d, *J =* 7.9), 7.48 (d, *J =* 7.4), 7.64 (2H, d, *J =* 7.5), 8.86 (1H, br. s.), 9.41 (1H, br. s.), 11.93–12.23 (1H, br. s.). ^13^C NMR (75 MHz, DMSO-d_6_): δ 13.1, 18.1, 19.0, 26.9, 27.2, 29.5, 32.2, 35.5, 43.5, 48.1, 48.8, 49.7, 62.3, 68.7, 114.7 (2C), 127.7 (2C), 129.2 (2C), 130.6 (2C), 131.1, 133.1, 138.1, 156.5, 173.8. Found, %: Cl 7.87. C_27_H_36_O_3_NCl. Calcd, %: Cl 7.74.

#### 4.1.5. Methyl 3-(4-(4-(((1*R*,2*S*,4*R*)-1,7,7-trimethylbicyclo[2.2.1]heptan-2-ylamino)- methyl)phenethyl)phenyl)propanoate **7**

A solution of bromide **5** (1.18 mmol), amine *endo*-**6** (1.46 mmol), and *N*,*N*-diisopropylethylamine (2.01 mmol) in acetonitrile (7 mL) was refluxed for 2 h. Ethyl acetate (20 mL) and a 5% sodium hydroxide solution (10 mL) were added. The organic layer was separated, and the aqueous layer was extracted with ethyl acetate (2 × 10 mL). Combined organic layers were washed with water (10 mL) and brine (10 mL) and were dried over magnesium sulfate. The solvent was evaporated in vacuum, and the residue was purified by silica gel column chromatography using chloroform–methanol 100:1. White solid, 52% yield. M.p. 84.5–87.1 °C. HRMS for C_28_H_37_O_3_N^+^ *m/z* calcd. [M]^+^ 435.2768, found 435.2765. ^1^H NMR (300 MHz, CDCl_3_): δ = 7.41–7.33 (4H, m), 7.11 (2H, d, *J =* 8.6), 6.92–6.88 (2H, m), 5.02 (2H, s), 3.90–3.83 (1H, m), 3.77–3.70 (1H, m), 3.67 (3H, s), 2.94–2.83 (3H, m), 2.65–2.57 (2H, m), 2.22–2.09 (1H, m), 1.85 (1H, d.d.d, *J =* 4.2, 8.9, 12.6), 1.79–1.59 (2H, m), 1.34–1.13 (3H, m), 0.91–0.81 (10H, m). ^13^C NMR (101 MHz, CDCl_3_): δ 14.3, 18.7, 19.8, 27.4, 28.4, 30.1, 35.9, 38.0, 45.0, 48.3, 48.8, 51.6, 52.8, 62.6, 69.9, 114.8 (2C), 127.5 (2C), 128.2 (2C), 129.2 (2C), 132.8, 135.4, 141.1, 157.3, 173.4.

#### 4.1.6. Methyl 3-(4-(4-(((1*S*,2*R*,4*R*)-1,7,7-trimethylbicyclo[2.2.1]heptan-2-yloxy)methyl)- benzyloxy)phenyl)Propanoate **9**

A mixture of borneol **8** (1.2 mmol), bromide **5** (242 mg, 0.8 mmol), diisopropylethylamine (490 μL, 2.4 mmol), and potassium iodide (1 mg, 0.03 mmol) was stirred in an inert atmosphere at 150 °С for 1 h. The mixture was cooled, 10 mL of ethyl acetate was added, and the obtained solution was washed successively with 2 mL of water and 2 mL of brine. The organic phase was separated and dried over magnesium sulfate. The latter was filtered off, and the solvent was evaporated under reduced pressure. The residue was purified by column chromatography (eluent: chloroform). White powder, yield 41%. M.p. 66.5–68.4 °C. [α]_D_^25^ +32 (c 0.137, CHCl_3_). HRMS for C_28_H_36_O_4_^+^ *m/z* calcd. [M]^+^ 436.2608, found 436.2620. IR (KBr, cm^−1^): ν 829, 1171, 1514, 1732. ^1^H NMR (400 MHz, CDCl_3_): δ 0.84 (3H, s), 0.86 (3H, s), 0.89–0.94 (3H, с), 1.10 (1H, d.d, *J =* 13.0, 3.2), 1.20–1.31 (2H, m), 1.63–1.78 (2H, m), 2.07–2.19 (2H, m), 2.60 (2H, t, *J =* 7.7), 2.90 (2H, t, *J =* 7.7), 3.66–3.73 (4H, m), 4.46 (1H, d, *J =* 12.2), 4.58 (1H, d, *J =* 12.2), 5.03 (2H, s), 6.90 (2H, d, *J =* 8.6), 7.11 (2H, d, *J =* 8.5), 7.34–7.42 (4H, m). ^13^C NMR (126 MHz, CDCl_3_): δ 14.0, 18.9, 19.8 (2C), 26.8, 28.3, 30.1, 35.9, 36.1, 45.1, 47.9, 49.3, 51.5, 69.9, 71.3, 84.4, 114.9 (2С), 127.4 (2С), 127.4 (2C), 129.2 (2C), 132.9, 136.0, 139.4, 157.4, 173.3.

#### 4.1.7. 4-(4-(Bromomethyl)benzyloxy)benzaldehyde **12**

A solution of hydroxyaldehyde **10** (620 mg 3.4 mmol) and potassium carbonate (1.30 g, 9.4 mmol) in 10 mL of acetone was refluxed with stirring for 1 h. The resulting solution was cooled to room temperature and decanted into a dropping funnel, and the volume of the solution was brought to 40 mL. Then, 1,4-Bis(bromomethyl)benzene **11** (3.58 g, 13.6 mmol) in acetone (150 mL) was added to the residue. With stirring, the previously obtained solution of hydroxyaldehyde **10** in acetone was added dropwise to a boiling solution of 1,4-bis(bromomethyl)benzene for 2 h. After all the solution was added, the reaction mixture was refluxed for another 2 h. The solution was cooled to room temperature, and the precipitate was filtered off. The precipitate was washed twice with 20 mL of chloroform. The combined filtrates were evaporated in a rotary evaporator, and the residue was recrystallized from acetone to isolate an excess of 1,4-bis-(bromomethyl)benzene. The mother liquor was evaporated, and the residue was purified by column chromatography (hexane–ethyl acetate 20:1 → 9:1). White solid, yield 62%. ^1^H NMR (400 MHz, CDCl_3_): δ 4.51 (2H, s), 5.14 (2H, s), 7.07 (2H, d, *J =* 8.6), 7.38–7.48 (4H, m), 7.84 (2H, d, *J =* 8.7), 9.89 (1H, s).

#### 4.1.8. Diethyl 2-(4-((4-Formylphenoxy)methyl)benzyl)malonate **13**

To an ice-cooled suspension of sodium hydride (76 mg, 1.9 mmol) in 5 mL of THF, diethyl malonate (500 μL, 3.3 mmol) was added dropwise with stirring. After 1 h, a solution of bromaldehyde **12** (477 mg, 1.6 mmol) in 2 mL of THF was added dropwise. After 2 h, the mixture was poured into 30 mL of ethyl acetate and washed through 10 mL of 2 M hydrochloric acid, 10 mL of water, and 10 mL of brine. The organic phase was dried with magnesium sulfate. The latter was filtered off, and the solvent was evaporated under reduced pressure. The residue was purified by column chromatography (hexane–ethyl acetate 4:1). Colorless liquid, yield 67%. HRMS for C_22_H_24_O_6_^+^ *m/z* calcd. [M]^+^ 384.1567, found 384.1564. IR (neat, cm^−1^): ν 1159, 1228, 1254, 1601, 1693, 1732, 1747. ^1^H NMR (500 MHz, CDCl_3_) δ 1.20 (6H, t, *J =* 7.1), 3.23 (2H, d, *J =* 7.7), 3.64 (1H, t, *J =* 7.9), 4.10–4.21 (4H, m), 5.11 (2H, s), 7.06 (2H, d, *J =* 8.7), 7.24 (2H, d, *J =* 8.0), 7.34 (2H, d, *J =* 8.0), 7.83 (2H, d, *J =* 8.7), 9.88 (1H, s). ^13^C NMR (126 MHz, CDCl_3_): δ 14.0, 34.3, 53.7, 61.5 (2C), 70.0, 115.1 (2C), 127.7 (2C), 129.2 (2C), 130.1, 131.9 (2C), 134.4, 138.2, 163.7, 168.7 (2C), 190.7.

#### 4.1.9. Ethyl 3-(4-((4-Formylphenoxy)methyl)phenyl)propanoate **14**

A solution of **13** (1.67 g, 4.3 mmol), sodium chloride (0.38 g, 6.5 mmol), and water (0.12 mL, 6.5 mmol) in 5 mL of dimethyl sulfoxide was refluxed for 4 h. After the completion of the reaction, the mixture was poured into 40 mL of ethyl acetate and washed successively with water (2 × 10 mL) and brine (10 mL). The organic phase was dried with magnesium sulfate, which was next filtered off, and the solvent was evaporated under reduced pressure. The residue was purified by column chromatography (eluent: hexane–ethyl acetate 4:1). White solid, yield 78%. M.p. 36.1–36.4 °С. HRMS for C_19_H_20_O_4_^+^ *m/z* calcd. [M]^+^ 312.1356, found 312.1360. IR (KBr, cm^−1^): ν 835, 1163, 1255, 1578, 1603, 1689, 1718, 1728. ^1^H NMR (400 MHz, CDCl_3_): δ 1.23 (3H, t, *J =* 7.1), 2.62 (2H, t, *J =* 7.8), 2.96 (2H, t, *J =* 7.7), 4.12 (2H, q, *J =* 7.1), 5.10 (2H, s), 7.06 (2H, d, *J =* 8.7), 7.24 (2H, d, *J =* 8.1), 7.35 (2H, d, *J =* 8.1), 7.79–7.86 (2H, m), 9.87 (1H, s). ^13^C NMR (126 MHz, CDCl_3_): δ 14.2, 30.6, 35.7, 60.4, 70.1, 115.1 (2C), 127.8 (2C), 128.7 (2C), 130.1, 131.9 (2C), 133.8, 140.8, 163.7, 172.7, 190.7.

#### 4.1.10. Ethyl 3-(4-((4-(((1*R*,2*R*,4*R*)-1,7,7-Trimethylbicyclo[2.2.1]heptan-2-ylamino)methyl)- phenoxy)methyl)phenyl)propanoate **15**

Sodium triacetoxyborohydride (406 mg, 1.92 mmol) was added to a mixture of aldehyde **14** (325 mg, 1.0 mmol), amine *exo*-**6** (1.3 mmol), and acetic acid (58 μL, 1.0 mmol) in 10 mL of methylene chloride. The reaction mixture was stirred at room temperature for 5 h. Then, 10 mL of chloroform was added, and the resulting solution was washed sequentially with 5 mL of 5% sodium hydroxide, 5 mL of water, and 5 mL of brine and was dried over anhydrous magnesium sulfate. The latter was filtered off, and the solvent was evaporated under reduced pressure. The residue was purified by column chromatography (eluent: chloroform–methanol 20:1). Colorless oil, yield 77%. [α]_D_^25^ −52 (c 0.137, CHCl_3_). HRMS for C_29_H_39_O_3_N^+^ *m/z* calcd. [M]^+^ 449.2925, found 449.2926. IR (neat, cm^−1^): ν 822, 1173, 1240, 1510, 1736. ^1^H NMR (500 MHz, CDCl_3_): δ 0.87 (3H, s), 0.93 (3H, s), 1.08–1.15 (5H, m), 1.17–1.22 (1H, m), 1.25–1.30 (3H, m), 1.50–1.78 (5H, m), 2.65 (3H, q, *J =* 7.6), 2.99 (2H, t, *J =* 7.7), 3.59 (1H, d, *J =* 13.1), 3.75 (1H, d, *J =* 13.1), 4.17 (2H, q, *J =* 7.2), 5.04 (2H, s), 6.95 (2H, d, *J =* 7.0), 7.23–7.29 (4H, m), 7.38 (2H, d, *J =* 7.5). ^13^C NMR (126 MHz, CDCl_3_): δ 12.1, 14.1, 20.5, 20.5, 27.3, 30.6, 35.7, 36.8, 38.8, 45.3, 46.6, 48.3, 52.0, 60.2, 66.2, 69.8, 114.5 (2C), 127.6 (2C), 128.4 (2C), 129.0 (2C), 133.8, 135.1, 140.2, 157.6, 172.6.

### 4.2. Biological Experiments

Statistical analysis was performed by the Mann–Whitney *U* test. Data are shown as mean ± SEM. Data with *p* < 0.05 were considered statistically significant.

#### 4.2.1. Animals

Male C57BL/6 and AY mice weighing 22–25 g were obtained from the specific pathogen-free animal facility of the Institute of Cytology and Genetics SB RAS. The animals were kept under standard conditions with free access to water and feed in humidity-and-temperature–controlled rooms on a 12/12 h light-and-dark cycle. All manipulations with animals were carried out in strict accordance with the laws of the Russian Federation, a decree of the Ministry of Health of the Russian Federation no. 199n of 4 January 2016, and Directive 2010/63/EU of the European Parliament and of the Council of the European Union of 22 September 2010 on the protection of animals used for scientific purposes. The protocol of the animal experiment was approved by the Ethics Committee of N.N. Vorozhtsov Institute of Organic Chemistry SB RAS (protocol no. P-12-122019-14).

#### 4.2.2. The OGTT

The test was performed on mice after a 12 h fast (*n* = 6 in each group). Oral glucose loading (2.5 g/kg) was done in all the groups of mice. Prior to dissolution in water, all compounds were mixed with two drops of Tween 80. VIL tablets (Galvus, Novartis Farmaceutica SA, Barcelona, Spain) were dissolved in water. The tested compounds were given by oral gavage 30 min prior to the glucose load. Blood glucose was quantified with a ONE TOUCH Select blood glucose meter (LIFESCAN Inc., Milpitas, CA, USA) before dosing (time 0) and at 30, 60, 90, and 120 min after the glucose load. The area under the glycemic curve was calculated using Tai’s model [35].

#### 4.2.3. The Design of the Experiment on AY Mice

The mice were fed standard chow plus lard and cookies ad libitum for 60 days until they gained body weight more than 35 g. After that, the following groups were set up: 1) AY mice + compound **1** 30 mg/kg, 2) AY mice + compound **3** 30 mg/kg, 3) AY mice + VIL 30 mg/kg, 4) AY mice + vehicle (water + 2 drops of Tween 80), and 5) untreated C57BL/6 mice. Each group consisted of 8 animals. The diet stayed the same until the end of the experiment. All compounds were administered by oral gavage once a day. OGTT was performed on the first and 14th days of the experiment. At the end of the experiment (16 days), blood was drawn for biochemical assays, and the liver was excised for histological evaluation.

#### 4.2.4. Biochemical Assays

After 16 days of treatment, mice were euthanized, blood was collected from the jugular vein, and serum was separated by centrifugation at 1640× *g* for 15 min. Serum total cholesterol, total triglycerides, high-density and low-density lipoprotein cholesterol, and glucose levels were quantified in all groups using standard diagnostic kits (Vector-Best, Novosibirsk, Russia) and a Stat Fax 3300 spectrophotometer (Awareness Technology Inc., Palm City, FL, USA).

#### 4.2.5. Histological Examination

Each liver was fixed in 10% neutral buffered formalin for 7 days, then standard dehydration in ascending ethanol concentrations and xylene was carried out, and the specimens were embedded in paraffin on an AP 280 workstation using Histoplast (Thermo Fisher Scientific, Waltham, MA, USA) with a melting point of 58 °C. Tissue slices with a thickness of 4.5 μm were prepared on a rotational NM 335E microtome with disposable interchangeable blades. The slices were stained with periodic acid–Schiff, hematoxylin and eosin, and orange G and examined under a light microscope at a magnification of ×100–200.

### 4.3. Computational Analyses

The prediction of activity towards the biotargets that determine hypoglycemic activity of phenylpropanoic acid derivatives was performed by the 2D (two-dimensional) structural similarity method using the Microcosm BioS v20.6.6 system (June 2020), which includes the original QSAR database containing verified and processed information on the chemical structure and activity levels of 625,888 known compounds studied by the international scientific community for 11,509 types of targeted biological activity [19]. Preprocessing of structural information was carried out in the IT Microcosm software [20]. In Microcosm BioS QSAR-base, an ordered series of values of each type of activity, Act, is divided into deciles and each decile range is assigned (on a 10-point scale) an ordinal index of an activity level, Ind_Act_, which takes the following values: +5 (very highly active); +4 (greatly active); +3 (highly active); +2 (significantly above moderate); +1 (above moderate); 0 (moderate); −1 (below moderate); −2 (significantly below moderate); −3 (weakly active); −4 (very weakly active); −5 (inactive). For each analyzed compound i for each type of predicted activity, Act, using the QL-modified Tanimoto similarity coefficient [20,22], 10 nearest neighbors j were calculated with indices of the activity level Ind_Act,i,j_. The average value of these indices is a metric of the expected level of this activity Ind_Mean,i_ for compound i. In this case, for each compound i, the average similarity coefficient T_Mean,i_ was computed in relation to 10 standards; this metric reflects the degree of the presence of this type of activity in the untested compound, whereas indicator Ind_Mean,i_ characterizes the possible level of this activity.

## 5. Conclusions

In this work, we synthesized four bornyl derivatives of *p*-(benzyloxy)phenylpropionic acid and analyzed (by in silico methods) the profile of their effects on biotargets relevant to hypoglycemic action. The computational prediction showed that the studied substances are most likely bifunctional multitarget hypoglycemic compounds whose mechanism of action is based on a reduction in insulin resistance and a strong incretin-mimetic effect. Biological experiments confirmed the calculated data. The two active substances selected in the OGTT yielded different results in C57BL/6Ay mice, which are characterized by impaired carbohydrate and lipid metabolism. Even though compounds **1** and **3** exert incretin-mimetic action in normal mice and have similar chemical structures, they have different effects under the conditions of lipid and carbohydrate metabolism disorders and fatty degeneration of the liver. Compound **3** reduces insulin resistance to a greater extent and restores liver function to a lesser extent, whereas compound **1** almost completely normalizes liver function and lipid metabolism but only indirectly reduces insulin resistance. All the findings indicate a difference between the studied substances in the targets of their pharmacological action.

These results mean that bornyl derivatives of *p*-(benzyloxy)phenylpropionic acid hold promise as new therapeutic agents for diabetes mellitus due to their hypoglycemic and lipid-normalizing properties.

## Figures and Tables

**Table 1:** Biotargets corresponding to possible hypoglycemic activities of the phenylpropanoic acid derivatives ^1^.

Target Name ^2^	Protein Symbol ^3^	UniProt ^4^	Ligand ^5^
Cleavage of poly- and oligosaccharides			
Pancreatic alpha-amylase	AMY2A	P04746	inhibitor
Maltase-glucoamylase, intestinal	MGAM	O43451	inhibitor
Insulinogens			
Sulfonylurea receptor 1	SUR1	Q09428	inhibitor
Incretin mimetics			
Dipeptidyl peptidase 4	DPP4	P27487	inhibitor
Gastric inhibitory polypeptide receptor	GIPR	P48546	agonist
Glucagon-like peptide receptor 1	GLP1R	P43220	agonist
Trace amine-associated receptor 1	TAAR1	Q96RJ0	agonist
Insulin resistance			
Free fatty acid receptor 1 (GPR40)	FFAR1	O14842	agonist
Free fatty acid receptor 2 (GPR43)	FFAR2	O15552	agonist
Free fatty acid receptor 3 (GPR41)	FFAR3	O14843	agonist
Free fatty acid receptor 4 (GPR120)	FFAR4	Q5NUL3	agonist
Peroxisome proliferator-activated receptor alpha	PPARA	Q07869	agonist
Peroxisome proliferator-activated receptor delta	PPARD	Q03181	agonist
Peroxisome proliferator-activated receptor gamma	PPARG	P37231	agonist
Protein-tyrosine phosphatase 1B (PTP1B)	PTPN1	P18031	inhibitor
Gluconeogenesis and glycogenolysis			
Glycogen phosphorylase, liver form	PYGL	P06737	inhibitor
Glycogen phosphorylase, muscle form	PYGM	P11217	inhibitor
Glucagon	GCG	P01275	inhibitor
Glucagon receptor	GCGR	P47871	inhibitor
Phosphoenolpyruvate carboxykinase, cytosolic	PCK1	P35558	inhibitor
Fructose-1,6-bisphosphatase 1	FBP1	P09467	inhibitor
Glycolysis and glycogen synthesis			
Pyruvate dehydrogenase kinase 4	PDK4	Q16654	activator
6-phosphofructokinase, muscle type	PFKM	P08237	activator
Glucose transport			
Sodium/glucose cotransporter 2	SGLT2	P31639	inhibitor

^1^ The biotargets are grouped by mechanism of action. ^2^ A common name for a biotarget. ^3^ An abbreviated name of a biotarget (matches the symbol of the gene for expressed protein). ^4^ A unique protein identifier in UniProtKB [19]. ^5^ A type of ligand that exerts hypoglycemic action.

**Table 2:** In silico-predicted relevance of biotargets and corresponding hypoglycemic activity of bornyl derivatives ^1^.

Target Name	Protein Symbol	Ind_Mean_^1^	T_Mean_^2^
Peroxisome proliferator-activated receptor delta	PPARD	**2.93**	**0.624**
Free fatty acid receptor 1 (GPR40)	FFAR1	**1.87**	**0.684**
Dipeptidyl peptidase 4	DPP4	**1.86**	**0.478**
Glycogen phosphorylase, liver form	PYGL	**1.70**	**0.460**
Protein-tyrosine phosphatase 1B (PTP1B)	PTPN1	**1.59**	**0.418**
Free fatty acid receptor 4 (GPR120)	FFAR4	**1.58**	**0.651**
Pancreatic alpha-amylase	AMY2A	**1.57**	0.195
6-phosphofructokinase, muscle type	PFKM	**1.00**	0.230
Gastric inhibitory polypeptide receptor	GIPR	***0.35***	**0.426**
Peroxisome proliferator-activated receptor alpha	PPARA	***0.33***	**0.627**
Glucagon-like peptide receptor 1	GLP1R	***0.00***	0.181
Pyruvate dehydrogenase kinase 4	PDK4	***0.00***	0.145
Trace amine-associated receptor 1	TAAR1	−0.18	**0.441**
Maltase-glucoamylase, intestinal	MGAM	−0.25	0.118
Peroxisome proliferator-activated receptor gamma	PPARG	−0.28	**0.586**
Glycogen phosphorylase, muscle form	PYGM	−0.33	0.381
Sulfonylurea receptor 1	SUR1	−0.50	**0.479**
Free fatty acid receptor 3 (GPR41)	FFAR3	−0.75	0.194
Glucagon receptor	GCGR	−0.76	**0.430**
Sodium/glucose cotransporter 2	SGLT2	−1.31	**0.491**
Fructose-1,6-bisphosphatase 1	FBP1	−1.45	0.384
Free fatty acid receptor 2 (GPR43)	FFAR2	−1.45	**0.459**
Glucagon	GCG	−1.65	**0.486**
Phosphoenolpyruvate carboxykinase, cytosolic	PCK1	−2.50	0.228

^1^ The average index of the expected activity level. Indices for highly important targets (strongly explaining the hypoglycemic activity) are bold; indices for medium-importance targets are bold and italic; other targets are defined as low-importance targets. ^2^ The average QL-modified Tanimoto similarity coefficient [20,22]. The values corresponding to high similarity of predicted and reference structures are highlighted in bold. Table 3 presents a profile of calculated activity levels for each of the four studied substances in relation to 24 analyzed biotargets.

**Table 3:** Profiles of targeted hypoglycemic activities of bornyl derivatives **1**–**4**.

Target Name	1	2	3	4
Peroxisome proliferator-activated receptor delta	**2.70**	**2.50**	**3.80**	**2.70**
Free fatty acid receptor 1 (GPR40)	**1.40**	**1.60**	**3.08**	**1.40**
Dipeptidyl peptidase 4	**2.00**	**2.54**	***0.89***	**2.00**
Glycogen phosphorylase, liver form	**1.70**	**1.70**	**1.70**	**1.70**
Protein-tyrosine phosphatase 1B (PTP1B)	**1.79**	**1.71**	**1.08**	**1.79**
Free fatty acid receptor 4 (GPR120)	**1.70**	**1.90**	**1.00**	**1.70**
Pancreatic alpha-amylase	**1.40**	**1.90**	**1.58**	**1.40**
6-phosphofructokinase, muscle type	**1.00**	**1.00**	**1.00**	**1.00**
Gastric inhibitory polypeptide receptor	***0.32***	***0.51***	***0.27***	***0.32***
Peroxisome proliferator-activated receptor alpha	***0.20***	−1.10	**2.00**	***0.20***
Glucagon-like peptide receptor 1	***0.00***	***0.00***	***0.00***	***0.00***
Pyruvate dehydrogenase kinase 4	***0.00***	***0.00***	***0.00***	***0.00***
Trace amine-associated receptor 1	−0.90	−1.10	**2.20**	−0.90
Maltase-glucoamylase, intestinal	−0.20	−0.20	−0.40	−0.20
Peroxisome proliferator-activated receptor gamma	−0.90	−0.90	**1.60**	−0.90
Glycogen phosphorylase, muscle form	−1.10	−0.80	**1.70**	−1.10
Sulfonylurea receptor 1	−0.50	−0.50	−0.50	−0.50
Free fatty acid receptor 3 (GPR41)	−0.75	−0.75	−0.75	−0.75
Glucagon receptor	−0.83	−0.66	−0.70	−0.83
Sodium/glucose cotransporter 2	−1.90	−2.10	***0.65***	−1.90
Fructose-1,6-bisphosphatase 1	−1.60	−0.70	−1.90	−1.60
Free fatty acid receptor 2 (GPR43)	−2.70	−2.20	**1.80**	−2.70
Glucagon	−1.73	−1.73	−1.40	−1.73
Phosphoenolpyruvate carboxykinase, cytosolic	−2.50	−2.50	−2.50	−2.50
Total sum of indices of activity levels	−1.40	0.12	**16.21**	−1.40
Sum of significant indices of activity levels	14.21	15.36	**24.36**	14.21

The table shows the average index of an expected activity level for each relation. Indices for highly important targets (strongly explaining the hypoglycemic activity) are bold; indices for medium-importance targets are bold and italic; other targets are defined as low-importance targets.

**Table 4:** A comparison of predictive estimates of the targeted activity of compounds **1–4**. The estimates were obtained by the method of similarity to standards and by the docking method.

Target	Compound	Ind ^1^	ΔE ^2^	Bin_Ind_^4^	Bin_ΔE_^5^
Peroxisome proliferator- activated receptor delta	**1**	2.70	−9.30	0	0
	**2**	2.50	−9.40	0	0
	**3**	3.80	−9.80	1	1
	**4**	2.70	−9.70	0	1
	Mean ^3^	2.93	−9.55		
Free fatty acid receptor 1 (GPR40)	**1**	1.40	−10.00	0	0
	**2**	1.60	−9.80	0	0
	**3**	3.08	−10.20	1	1
	**4**	1.40	−10.10	0	1
	Mean	1.87	−10.03		
Dipeptidyl peptidase 4	**1**	2.00	−9.10	1	1
	**2**	2.54	−9.10	1	1
	**3**	0.89	−8.80	0	0
	**4**	2.00	−9.10	1	1
	Mean	1.86	−9.03		
Spearman test R_Sp_				0.7143	
*p*				9.06 × 10^−3^	

^1^ The index of the level of activity, as calculated by the method of similarity to standards (see Table 3). ^2^ Docking energy, kcal/mol. ^3^ Average values for a biotarget and activity level indicator. ^4^ Binary assessment of the level of activity, as calculated by the method of similarity to standards. ^5^ A binary estimate of the level of activity, as calculated by the docking method.

**Table 5:** Blood biochemical parameters in treated AY mice at the end of the experiment.

Group	TC, mmol/L	TGs, mmol/L	HDL-C, mg/dL	LDL-C, mg/dL	Glu, mmol/L
C57BL/6 mice	1.59 ± 0.14	0.54 ± 0.04	82.66 ± 5.29	15.13 ± 2.02	5.55 ± 0.38
AY mice	2.59 ± 0.27 *	0.64 ± 0.03	99.33 ± 6.38	10.36 ± 1.49	7.15 ± 0.69 *
VIL, 30 mg/kg	3.26 ± 0.10 *	0.46 ± 0.04	109.67 ± 5.23 *	13.13 ± 0.95	8.41 ± 0.43 *
**1**, 30 mg/kg	1.79 ± 0.21 ^#^	0.58 ± 0.02	78.30 ± 5.73 ^#^	9.38 ± 1.25	5.16 ± 0.52 ^#^
**3**, 30 mg/kg	2.80 ± 0.25 *	0.64 ± 0.06	90.48 ± 8.6	10.78 ± 1.38	6.58 ± 1.06

* *p* ≤ 0.05 as compared with untreated C57BL/6 mice, ^#^ *p* < 0.05 as compared with untreated AY mice. TC: total cholesterol, TGs: triglycerides, HDL-C: high-density lipoprotein cholesterol, LDL-C: low-density lipoprotein cholesterol, Glu: glucose.

## Supplementary Materials

Supplementary materials can be found at https://www.mdpi.com/1424-8247/13/11/404/s1. Figure S1. 1H NMR spectrum of compound **2**. Figure S2. 13C NMR spectrum of compound 2. Figure S3. 1H NMR spectrum of compound **3**. Figure S4. APT spectrum of compound 3. Figure S5. 1H NMR spectrum of compound **4**. Figure S6. APT spectrum of compound 4. Figure S7. 1H NMR spectrum of compound **12**. Figure S8. 1H NMR spectrum of compound **13**. Figure S9. APT spectrum of compound **13**. Figure S10. 1H NMR spectrum of compound 14. Figure S11. APT spectrum of compound **14**. Figure S12. 1H NMR spectrum of compound 15. Figure S13. APT spectrum of compound **15.**
