# Supplementary material for: Bornyl Derivatives of p-(Benzyloxy)Phenylpropionic Acid: In Vivo Evaluation of Antidiabetic Activity
Source: Pharmaceuticals (Basel). 2020 Nov 19;13(11):404. doi: 10.3390/ph13110404 (PMC7699345; doi:10.3390/ph13110404)
Supplement: Supplementary file 1 [file pharmaceuticals-13-00404-s001.pdf]

# Bornyl derivatives of p-(benzyloxy)phenylpropionic acid: In vivo evaluation of antidiabetic activity

Sergey Kuranov, Olga Luzina, Mikhail Khvostov, DmitriyBaev, Darya Kuznetsova, NataliyaZhukova, Pavel Vassiliev, Andrey Kochetkov, Tatyana Tolstikova, NarimanSalakhutdinov

NMR SPECTRA OF NEW COMPOUNDS

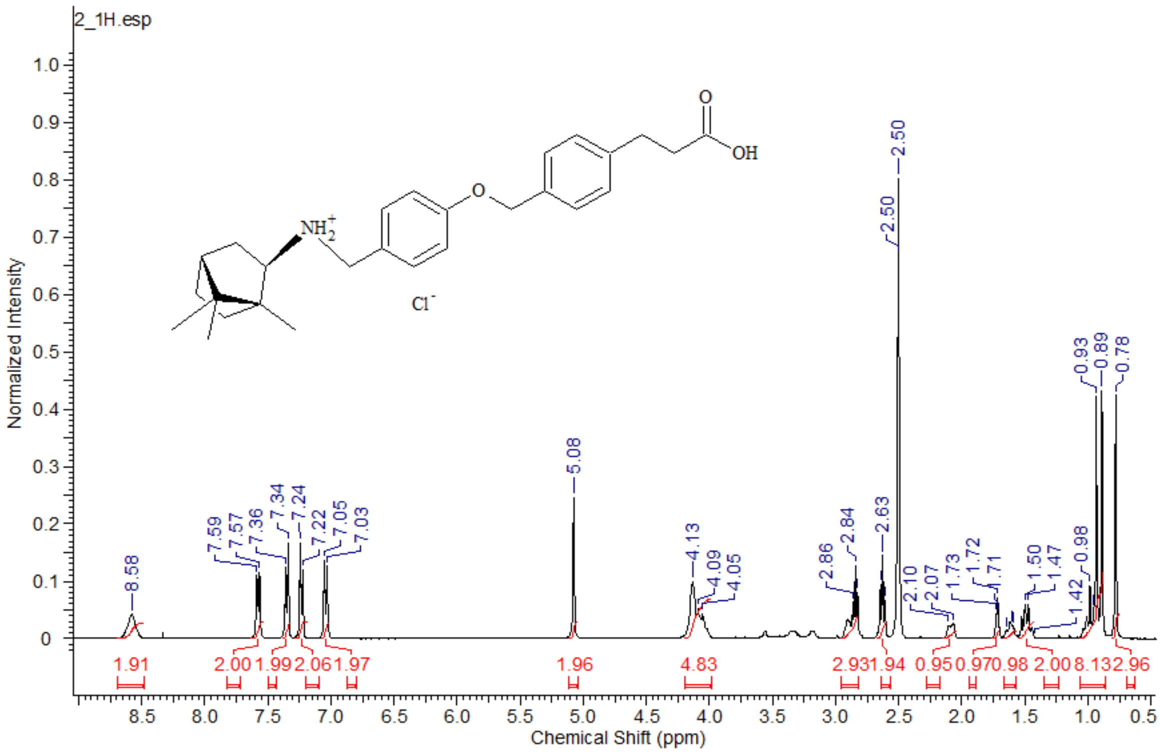


**Figure S1.** $^{1}H$ NMR spectrum of compound **2.**

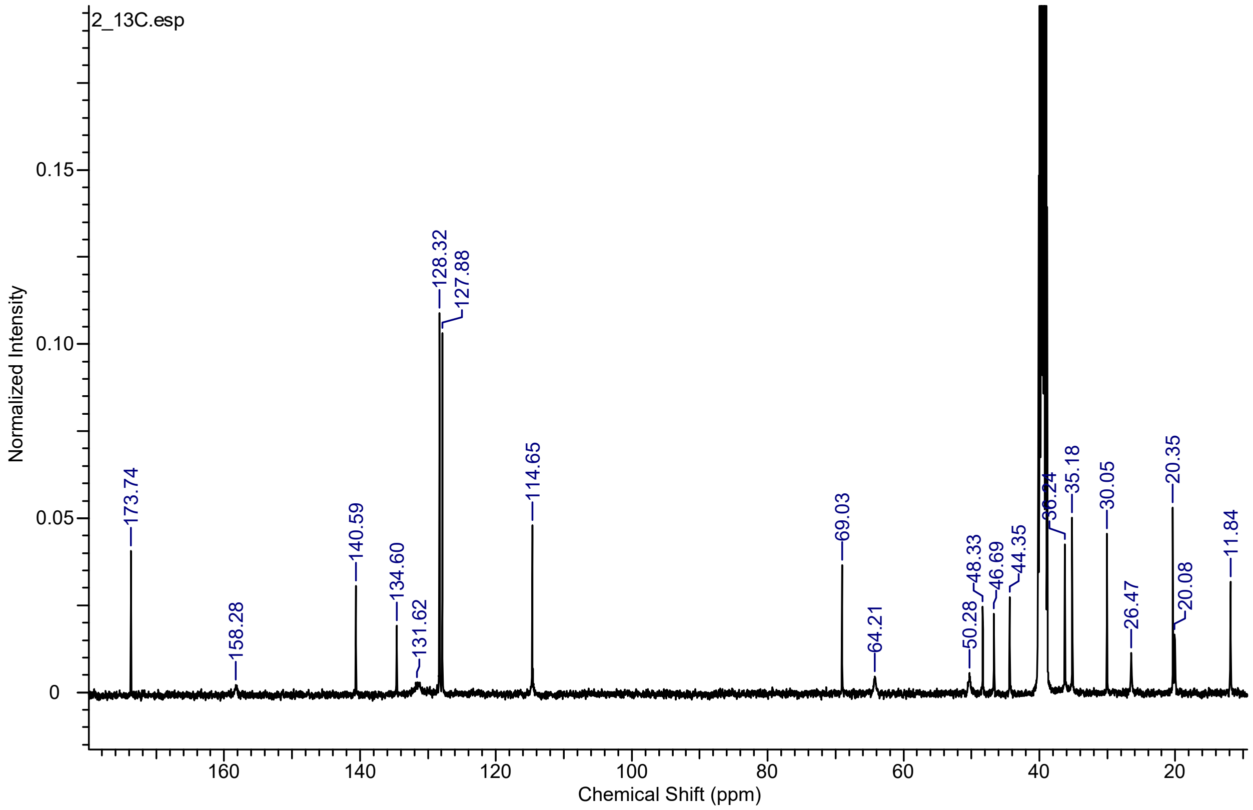


**Figure S2.** $^{13}C$ NMR spectrum of compound **2**

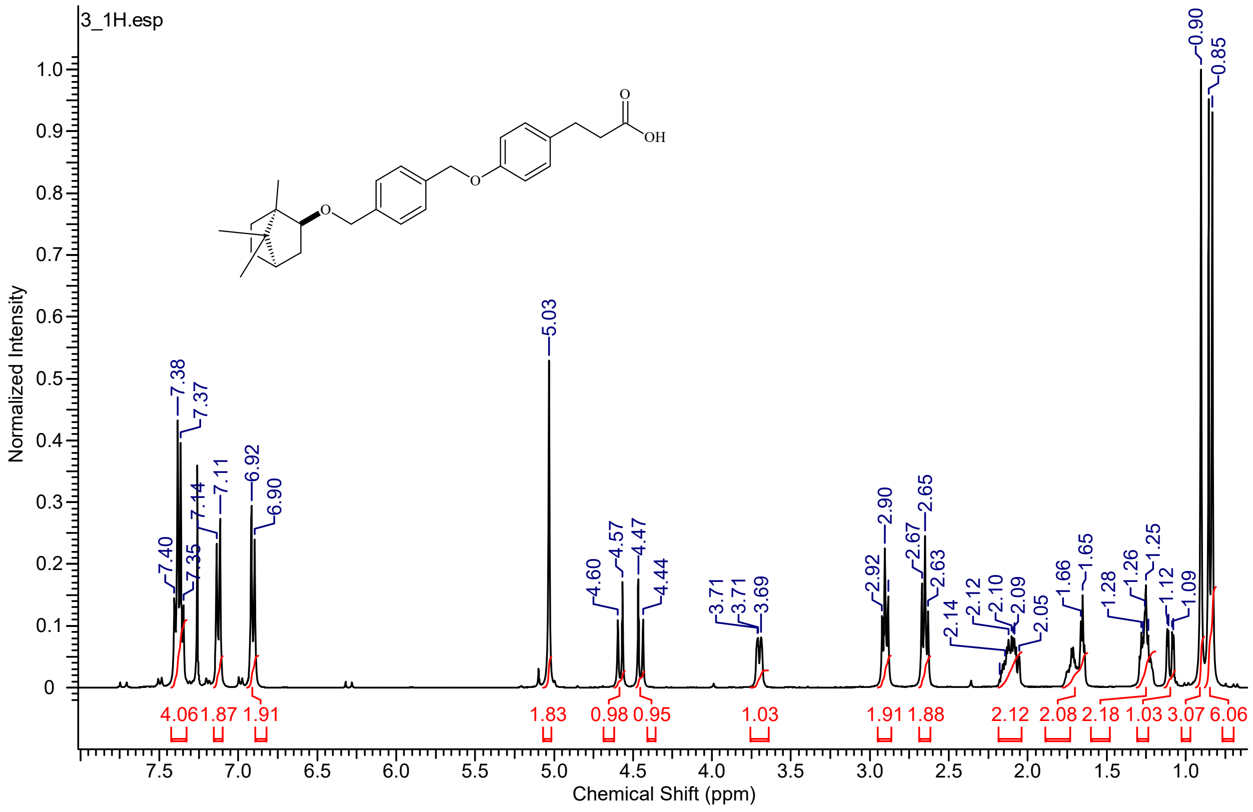


**Figure S3.**$^{1}$H NMR spectrum of compound **3**

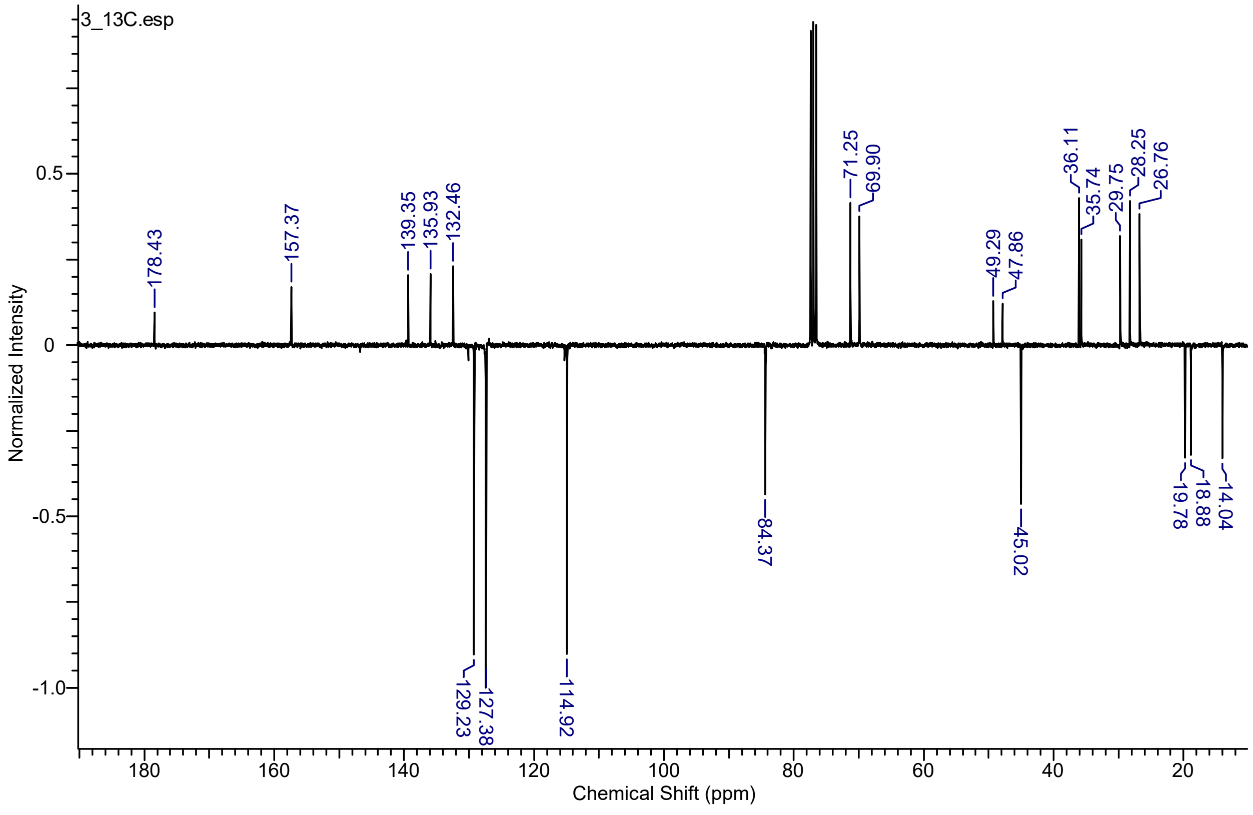


**Figure S4.**APT spectrum of compound**3**

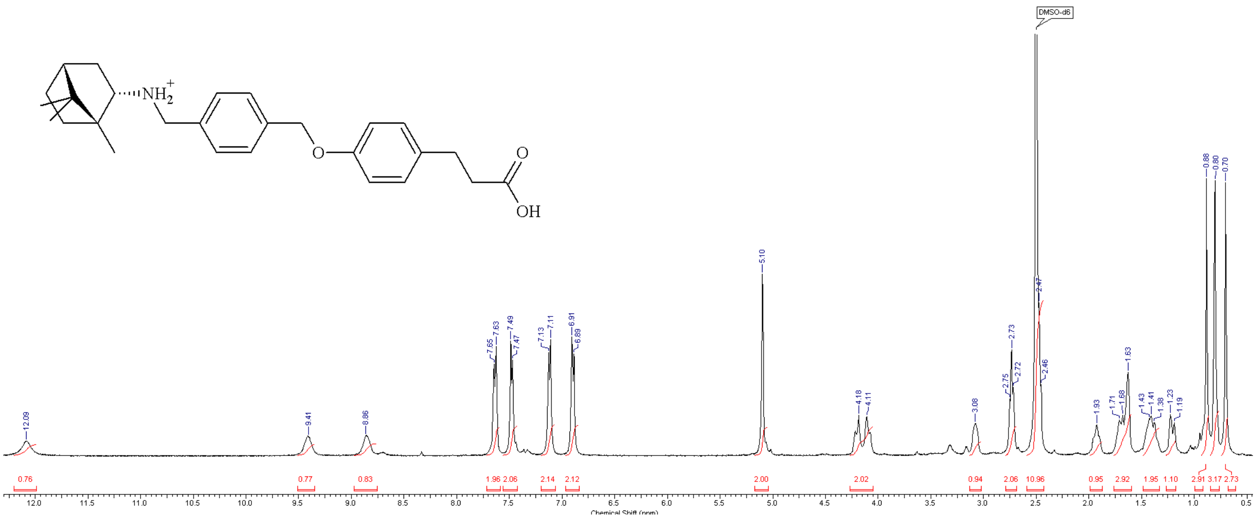


**Figure S5.**$^{1}$H NMR spectrum of compound **4**

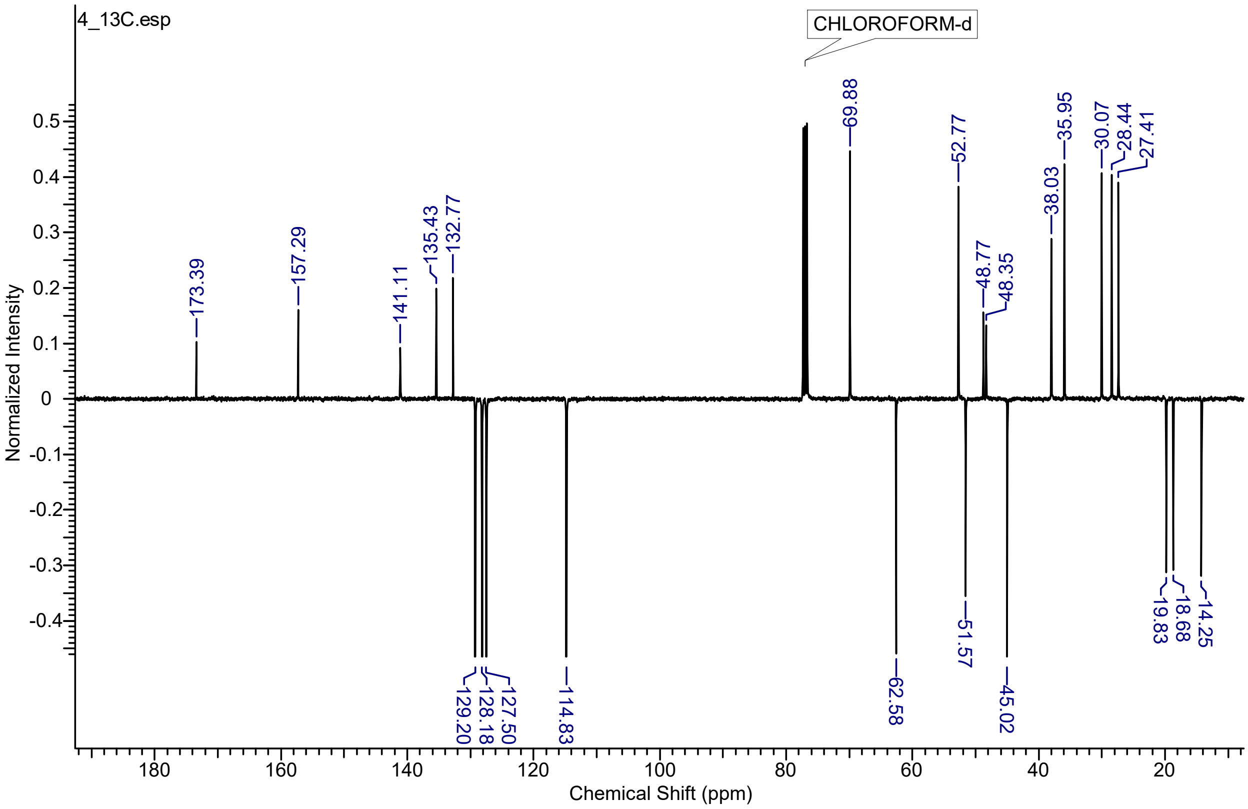


**Figure S6.**APT spectrum of compound**4**

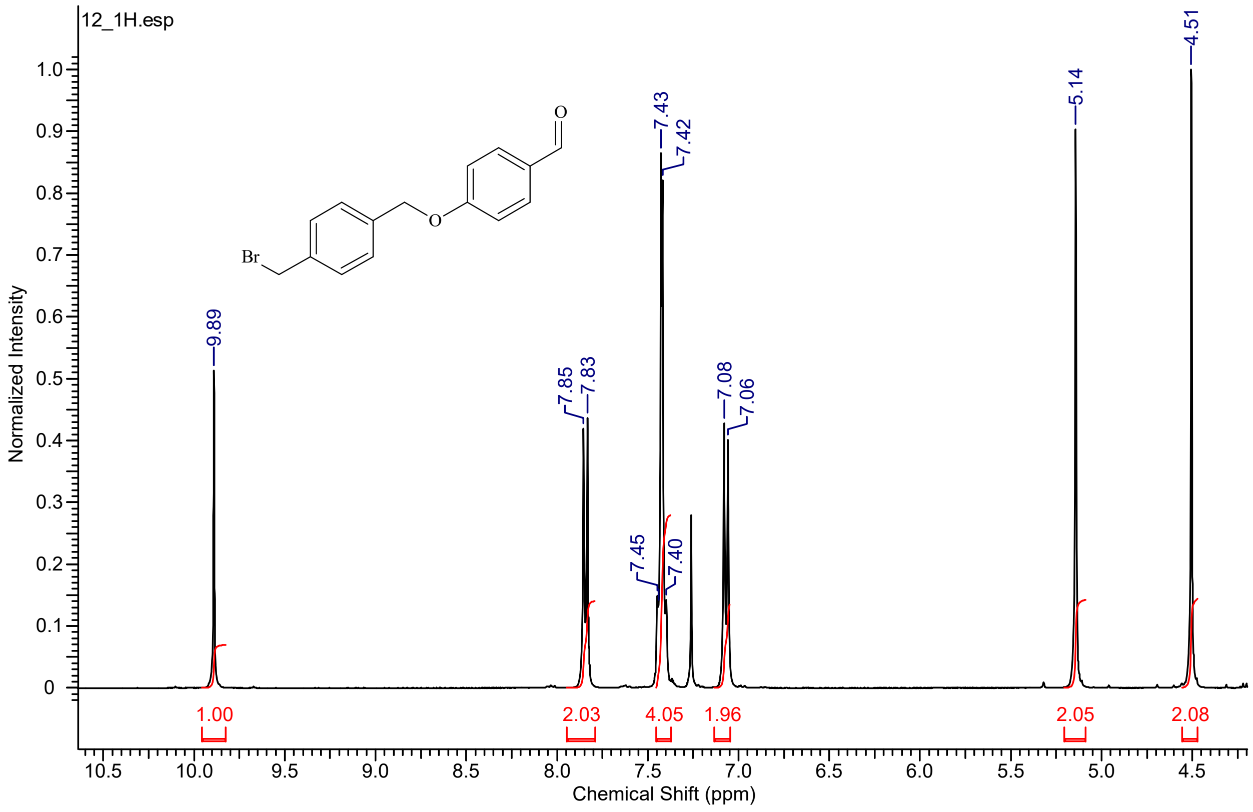


**Figure S7.** $^1$H NMR spectrum of compound **12**

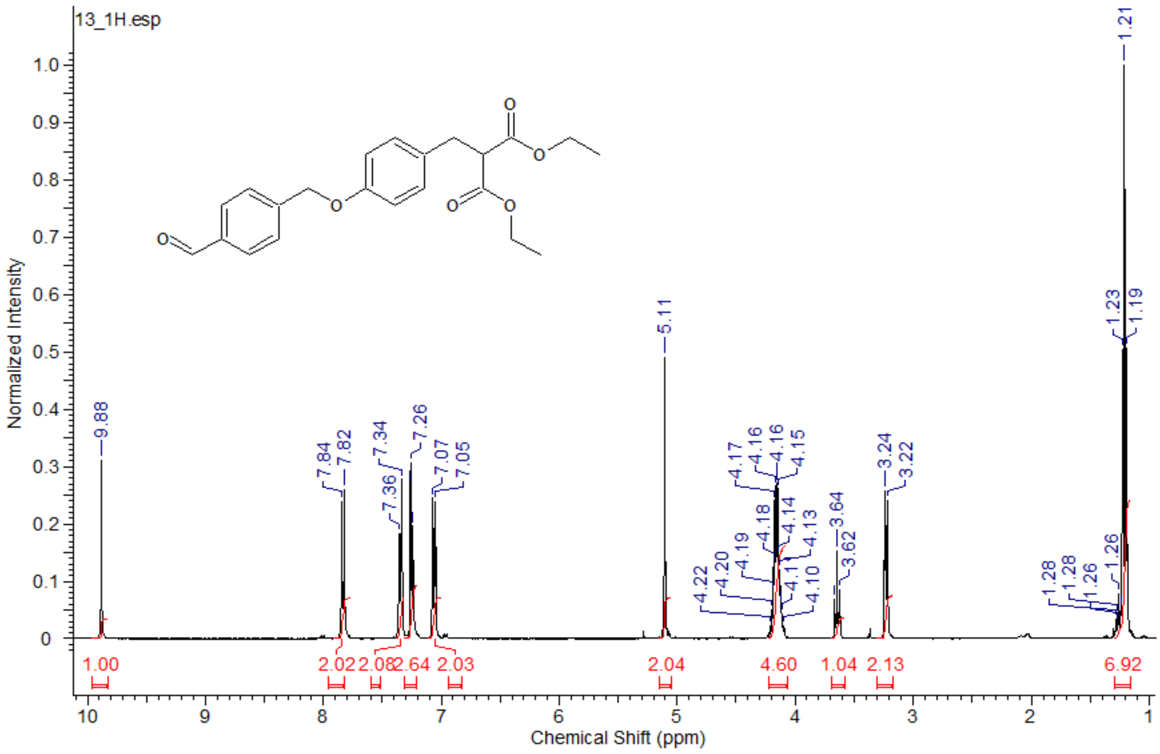


**Figure S8.**$^{1}$H NMR spectrum of compound **13**

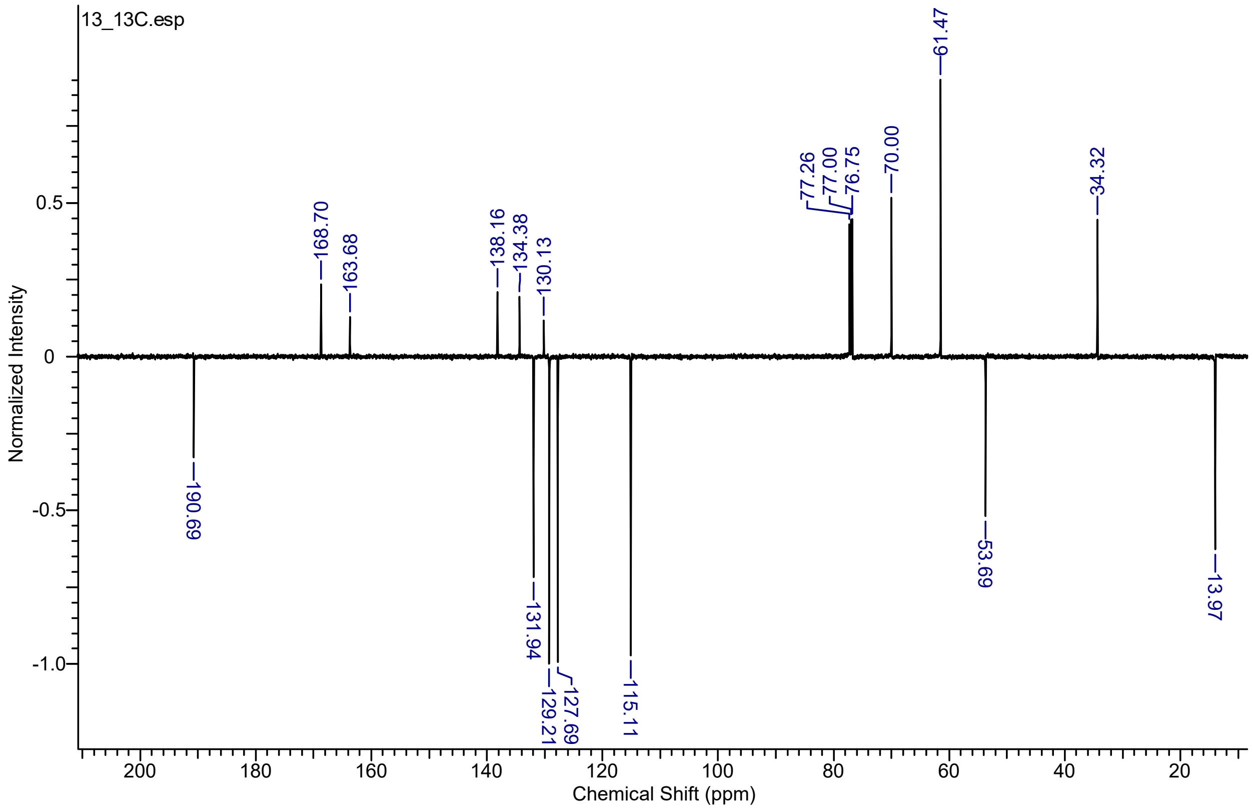


**Figure S9.**APT spectrum of compound**13**

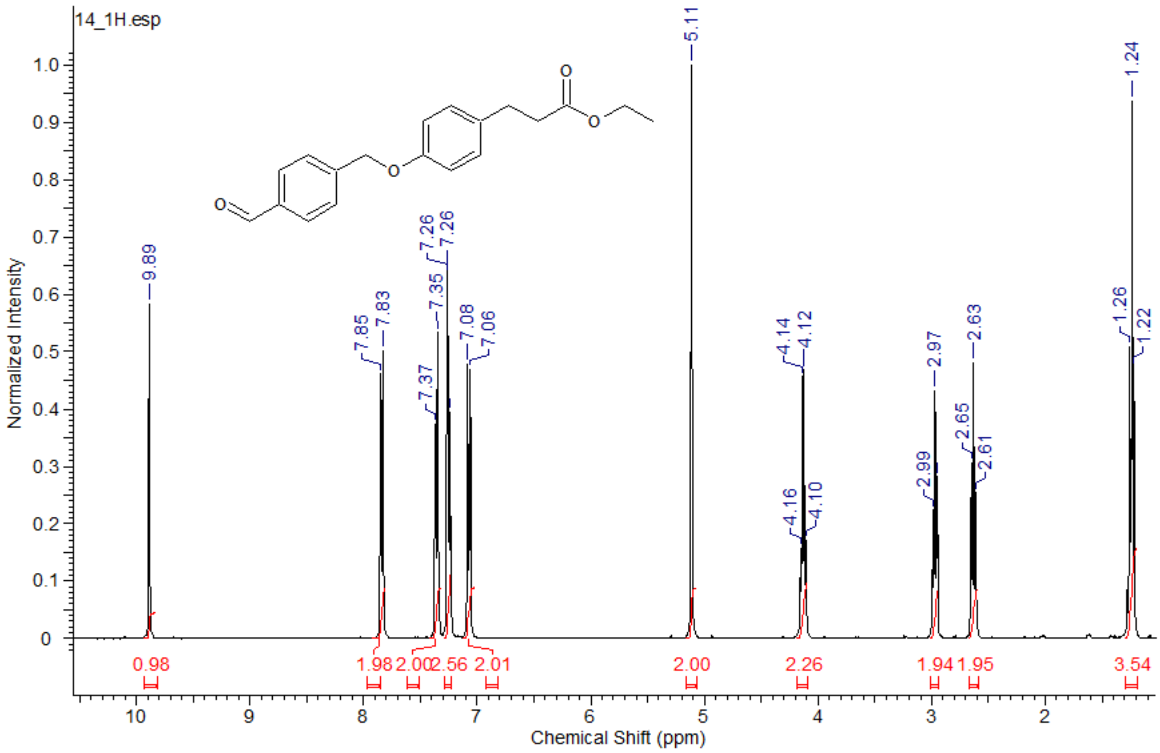


**Figure S10.**$^{1}$H NMR spectrum of compound **14**

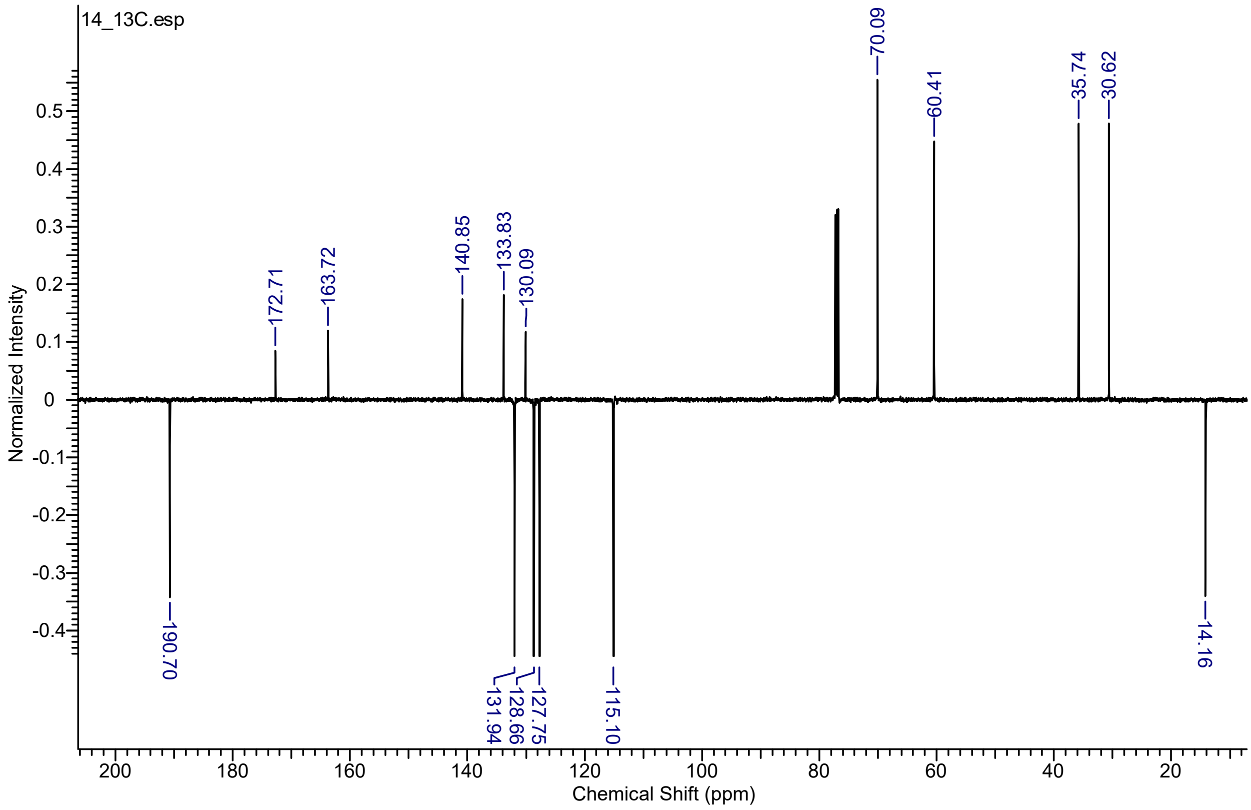


**Figure S11.**APT spectrum of compound**14**

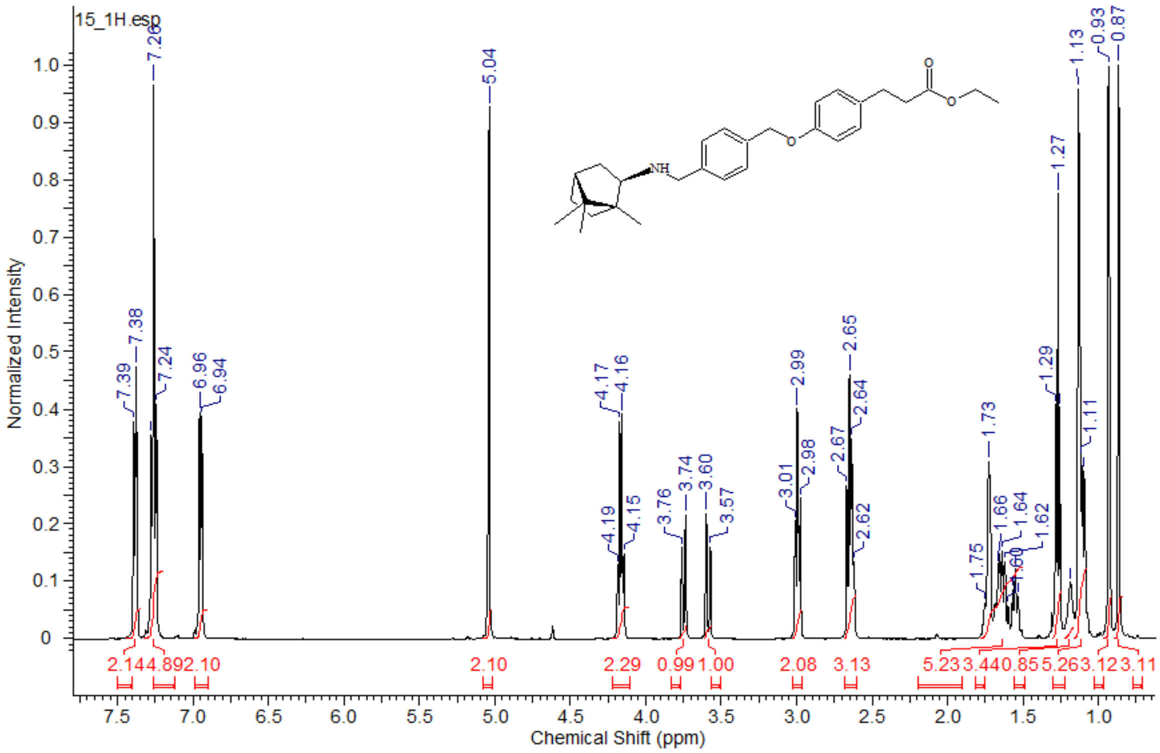

**Figure S12.**$^{1}$H NMR spectrum of compound **15**

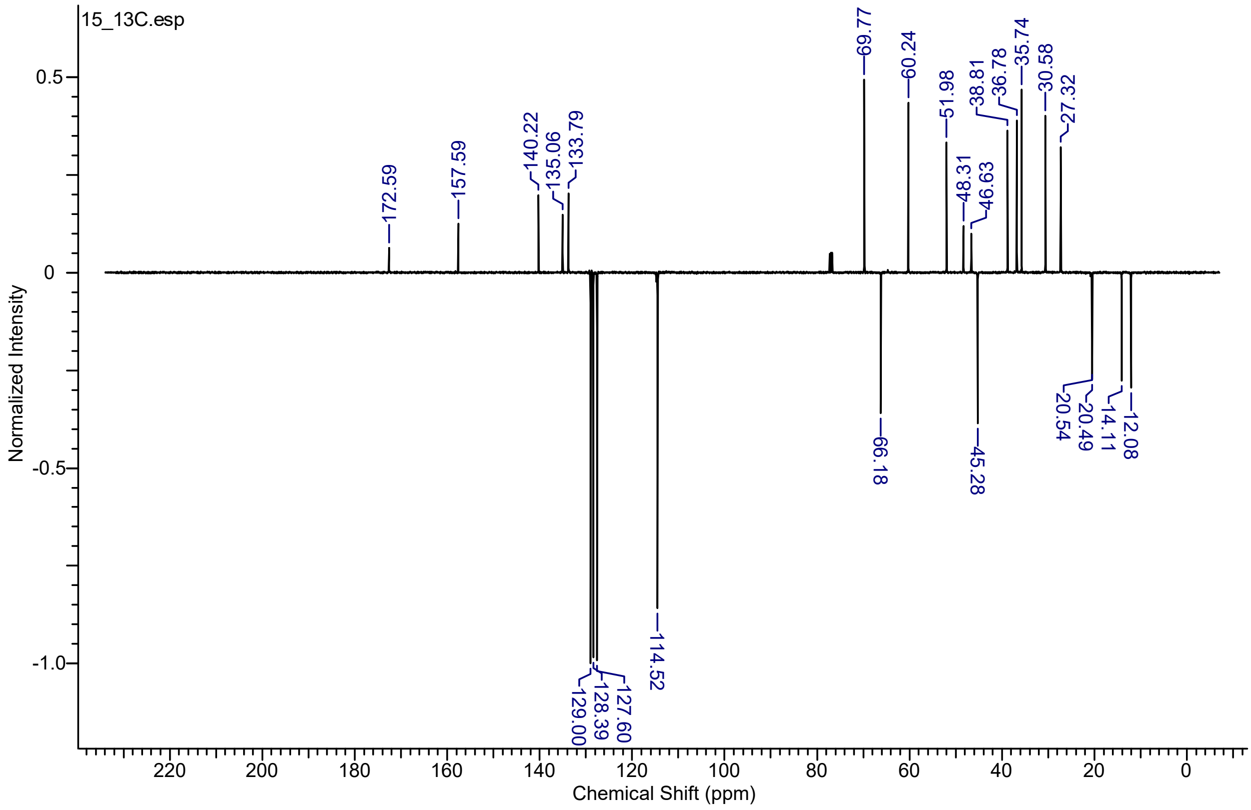

**Figure S13.**APT spectrum of compound**15**
